# YAP Fuels Cerebral Cavernous Malformations by Regulating KLF4 Transcriptional Activity and Protein Stability

**DOI:** 10.1002/mco2.71086

**Published:** 2026-10-23

**Authors:** Tingting Ye, Qi Zheng, Zonghao Hu, Haonan Fan, Zhongxing Sun, Xiya Li, Haomin Mo, Chengdong Chang, Yanhua Xu, Kai Wang, Dong Fang, Hai Song

**Affiliations:** ^1^ Life Sciences Institute Zhejiang University Hangzhou Zhejiang China; ^2^ Department of Cardiovascular Surgery the First Affiliated Hospital School of Medicine Zhejiang University Hangzhou Zhejiang China; ^3^ Department of Pathology The First Affiliated Hospital School of Medicine Zhejiang University Hangzhou Zhejiang China; ^4^ Department of Respiratory and Critical Care Medicine Center For Oncology Medicine the Fourth Affiliated Hospital of School of Medicine and International School of Medicine International Institutes of Medicine Zhejiang University Yiwu Zhejiang China; ^5^ Zhejiang Key Laboratory of Precision Diagnosis and Treatment for Lung Cancer Yiwu Zhejiang China; ^6^ The MOE Key Laboratory of Biosystems Homeostasis & Protection Zhejiang University Hangzhou Zhejiang China; ^7^ State Key Laboratory of Transvascular Implantation Devices Hangzhou China

## Abstract

Cerebral cavernous malformations (CCMs), which frequently predispose patients to strokes or seizures, primarily arise from endothelial specific loss‐of‐function mutations in the CCM genes, and occasionally accompanied by gain‐of‐function mutations in PIK3CA and MAP3K3. While the Hippo pathway is a well‐established regulator of tissue homeostasis, its emerging relevance in vascular pathology particularly its precise role in CCM pathogenesis remains poorly understood. In this study, we demonstrate that CCM gene deficiency suppresses endothelial Hippo signaling, as evidenced by diminished LATS activity, alongside concomitant elevation of YAP expression and nuclear translocation in both human and mouse CCM lesions. Notably, endothelial‐specific deletion of *Yap* mitigates CCM lesion formation, reinforces endothelial junctions, enhances inflammatory resistance, and decreases KLF4 protein levels in a CCM mouse model. Conversely, intracranial Adeno‐associated virus (AAV)‐mediated inhibition of LATS or activation of YAP promoted CCMs formation under *Ccm2* deficiency. Mechanistically, under the dual coordination of negative regulation by LATS and positive regulation by MAP3K3, YAP orchestrates KLF4 transcriptional activity and protein stability. This is achieved through a direct interaction with KLF4 that shields it from β‐TrCP‐mediated proteasomal degradation, thereby driving CCM progression. Collectively, our findings unveil a novel dimension to Hippo pathway regulation and underscore YAP as a pivotal driver of CCM pathogenesis.

## Graphical abstract

Cerebral cavernous malformation (CCM) gene complex deficiency dual‐regulates YAP activity via a “dual‐pronged” mechanism: relieving LATS‐mediated inhibition to license YAP nuclear translocation, and promoting MAP3K3‐mediated YAP phosphorylation to stabilize YAP–KLF4 association. This interaction shields KLF4 from β‐TrCP‐mediated ubiquitination and proteasomal degradation in endothelial cells, forming a pathogenic feed‐forward loop that accelerates vascular malformation.

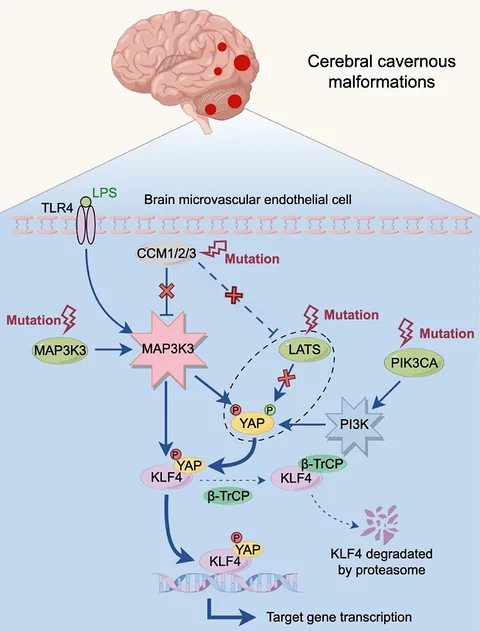

## 1 Introduction

Cerebral cavernous malformations (CCMs) represent central nervous system vascular anomalies affecting approximately 0.5% of the general population, frequently culminating in hemorrhagic strokes and seizures [1, 2]. CCMs typically originate from endothelial‐specific loss‐of‐function (LOF) mutations in one of the three CCM genes: *KRIT1* (*CCM1*), *CCM2*, or *PDCD10* (*CCM3*) [3]. These genetic aberrations trigger the constitutive activation of the MAP3K3–KLF2/4 signaling axis, which subsequently upregulates Rho signaling and ADAMTS proteolytic activity, thereby driving CCM pathogenesis [4]. Furthermore, external stimuli such as Toll‐like receptor 4 (TLR4) activation, as well as somatic gain‐of‐function (GOF) mutations, such as *MAP3K3* [c.1323C>G (p.Ile441Met)] and *PIK3CA^H1047R^*, have been established to accelerate lesion formation or growth [5, 6, 7, 8, 9]. Given the lack of effective pharmacological interventions targeting the MAP3K3–KLF2/4 cascade, coupled with the clinical challenge of multiple or surgically inoperable lesions, the identification of novel therapeutic strategies is urgently needed.

The Hippo signaling pathway represents an evolutionarily conserved signaling cascade essential for maintaining tissue homeostasis and orchestrating stress responses [10]. In mammals, this pathway is functionally centered on the core kinases LATS1/2, which directly phosphorylate and restrict the downstream transcriptional coactivators YAP/TAZ, thereby modulating gene expression programs governing cell proliferation, organ size control, and tissue regeneration [11]. The regulatory landscape of Hippo signaling is highly dynamic. Both PI3K and MAPK signaling pathways can suppress LATS activity to license the nuclear translocation and functional activation of YAP/TAZ [12, 13]. Consequently, aberrant deregulation of the Hippo pathway leads to uncontrolled cellular proliferation and tumorigenesis [14, 15]. Beyond its classical roles in tumor biology, emerging evidence from murine and zebrafish models has highlighted the critical requirement of Hippo signaling in regulating embryonic vasculogenesis, angiogenesis, and lymphangiogenesis [16, 17]. Notably, the loss of the tight junction protein ZO‐1, a foundational gatekeeper of endothelial barrier integrity, has been shown to stimulate YAP expression and activation in endothelial cells (ECs) [18]. Nevertheless, the precise pathological involvement of the Hippo pathway in vascular disorders characterized by aberrant EC proliferation, such as CCMs, as well as the underlying mechanistic underpinnings, remains poorly understood.

Mechanistically, LOF mutations within the CCM gene complex provoke the activation of its downstream kinase MAP3K3, which subsequently drives KLF2/4 expression via the MAP3K3–MEK5–ERK5 signaling axis [4]. Intriguingly, our previous study unveiled a direct interaction between MAP3K3 and YAP, culminating in specific YAP phosphorylation [19], which hints at a potential cross‐talk between these two cascades. Here, we aimed to elucidate the pathophysiological role of YAP in CCM mouse models with endothelial‐specific deletions of *Yap/Taz*. Our discoveries provide novel insights into the reciprocal molecular cross‐talk bridging Hippo–YAP signaling and CCM pathogenesis.

## 2 Results

### 2.1 YAP Protein Is Upregulated in CCM Disease and Plays a Pathogenic Role

To investigate the involvement of YAP in CCM pathogenesis, we first examined its expression in clinical samples. Immunofluorescence staining of 30 surgically resected human CCM lesions and 20 control tissues from peritumoral normal brain regions (resected CCM, *n* = 5; gliomas, *n* = 10; meningiomas, *n* = 5) revealed distinct YAP upregulation in CCM vascular ECs compared with controls (Figures 1A,B and S1A). Mirroring these human findings, endothelial deletion of *Ccm2* in mice substantially increased YAP expression within the cerebral vascular endothelium (Figure S1B).

**FIGURE 1:**
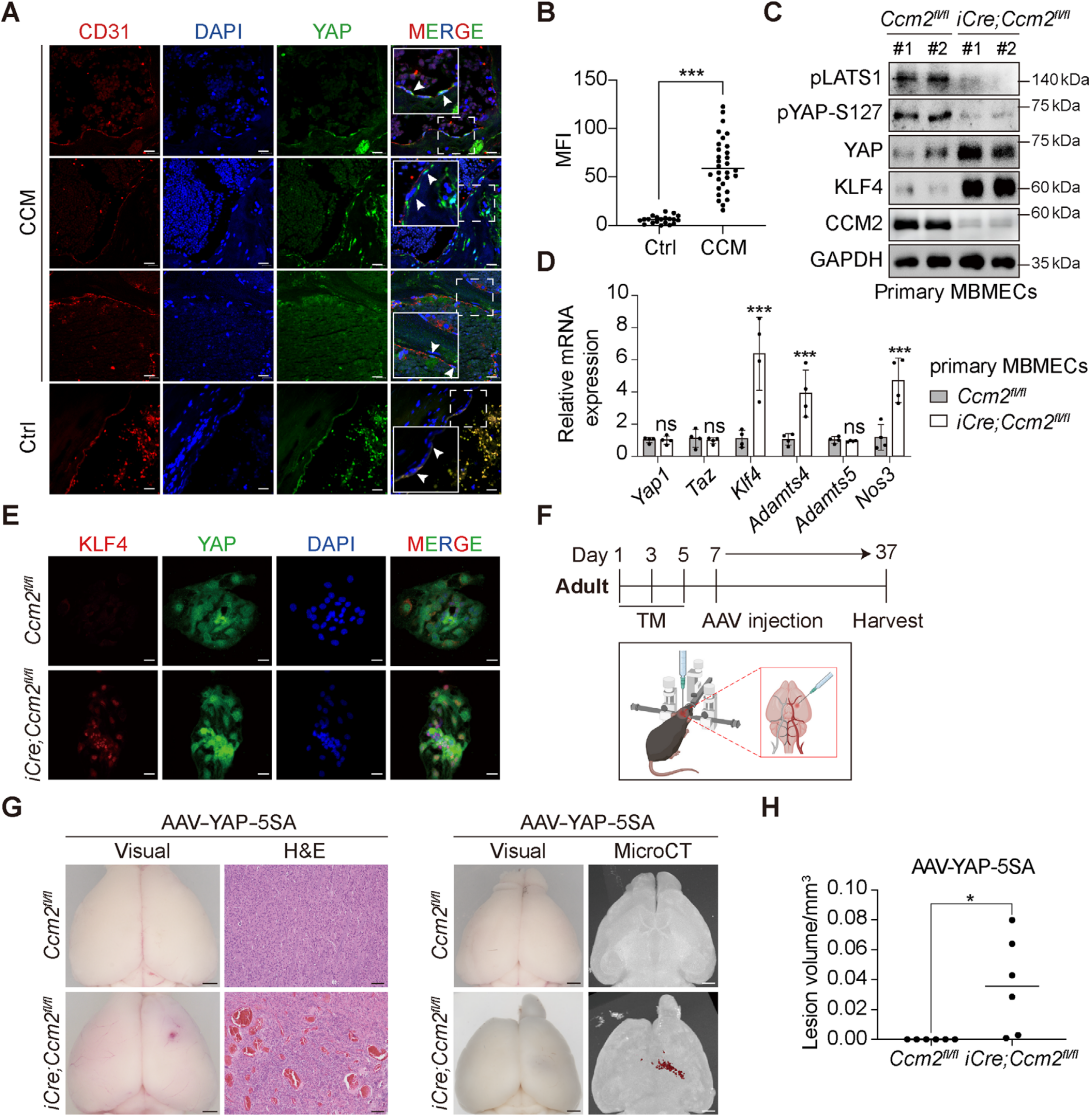
YAP protein is upregulated in CCM disease and plays a pathogenic role. (A) Representative immunofluorescence images of YAP (green) and the vascular endothelial marker CD31 (red) in surgically resected human CCM patients and control brain tissues. Arrows indicate endothelial YAP expression in CD31‐positive vascular ECs. Scale bars: 20 µm. (B) Quantitative analysis of endothelial YAP expression. Paraffin‐embedded sections were obtained from surgically resected tissues of 30 CCM patients and 20 control brain samples (peritumoral normal brain tissues of CCM, *n* = 5; gliomas, *n* = 10; meningiomas, *n* = 5). (C) Western blot analysis of YAP and KLF4 protein levels in *Ccm2*‐deficient primary MBMECs. (D) RT‐qPCR analysis of mRNA levels of *Klf4* and its downstream target genes in *Ccm2*‐deficient primary MBMECs (*n* = 4). (E) Immunofluorescence staining of YAP (green) and KLF4 (red) in primary MBMECs from adult *Ccm2^fl/fl^* and *iCre*;*Ccm2^fl/fl^* mice. Nuclei were counterstained with DAPI (blue). *n* = 3. Scale bars: 20 µm. (F) Schematic diagram illustrating intracranial AAV injection. (G) Representative gross morphology, micro‐CT, and H&E images of mouse brains collected 30 days postintracranial injection of AAV–YAP‐5SA into adult *Ccm2^fl/fl^* and *iCre*;*Ccm2^fl/fl^* mice. Scale bars: 1 mm (gross morphology and micro‐CT images); 50 µm (H&E images). (H) Quantification of lesion volumes from (G) by micro‐CT analysis (*n* = 6). Statistical analyses were performed using unpaired Student's *t*‐test (B and H) or one‐way ANOVA followed by Dunnett's multiple comparison test (D). Data are shown as mean ± SEM; *p < 0.05, **p < 0.01; n.s., not significant, *p* > 0.05.

To dissect the underlying mechanisms in vitro, we isolated primary mouse brain microvascular ECs (MBMECs) from adult *Ccm2^fl/fl^* and *iCre*;*Ccm2^fl/fl^* (*Cdh5^CreER^;Ccm2^fl/fl^*), where tamoxifen induces endothelial‐specific *Ccm2* ablation. Consistent with previous reports [4, 20], *Ccm2*‐deficient cells exhibited elevated expression of the CCM pathogenic marker *Klf4* (Figures 1C and S1C), with its downstream targets *Adamts4* and *Nos3* showing increased mRNA levels (Figure 1D). Intriguingly, *Ccm2* deletion suppressed Hippo signaling, as evidenced by decreased pLATS1 and pYAP–S127 levels alongside increased total YAP and TAZ protein levels, without altering *Yap* or *Taz* mRNA abundance (Figures 1C,D and S1C). Consistently, *Ccm2* loss triggered YAP nuclear localization in primary MBMECs (Figure 1E). This phenotype was fully recapitulated in bEnd.3 cells and human umbilical vein ECs subjected to siRNA‐mediated silencing or CRISPR/Cas9‐mediated depletion of *Ccm2*/*CCM2* (Figure S1D–I). Furthermore, *Ccm1* or *Ccm3* deficiency similarly elevated YAP protein levels in bEnd.3 cells (Figure S1J). Collectively, these findings indicate that YAP activation is a conserved consequence of CCM complex disruption.

To test whether YAP directly drives CCM pathogenesis, we overexpressed YAP and TAZ in bEnd.3 cells, which significantly induced KLF4 expression (Figure S2A). Next, we delivered a constitutively active YAP (5SA) mutant specifically to the endothelium using an AAV–BR1 vector. Because endothelial CCM deletion alone is insufficient to trigger brain hemorrhages in adult mice [4 ^,^ 9], we intracranially injected YAP–5SA AAV–BR1 into adult *iCre;Ccm2^fl/fl^* mice primed with tamoxifen to establish a disease‐sensitized state (Figure 1F). Remarkably, gross morphology, hematoxylin–eosin (H&E) staining, and micro‐CT analysis revealed characteristic CCM‐like hemorrhagic lesions in *Ccm2*‐deficient adult brains following YAP activation (Figures 1G,H and S2B). These lesions routinely developed within 1 month postinjection, localizing to the injection site and the highly vascularized olfactory bulb, whereas control mice remained lesion free (Figure 1G,H). Successful viral transduction was confirmed via immunohistochemical staining of the AAV‐tagged proteins (Figure S2C). Collectively, these data demonstrate that YAP activation exacerbates *Ccm2* deficiency‐induced CCM disease.

### 2.2 Endothelial Yap Deletion Alleviates CCM Progression

Unlike in adult mice, endothelial deletion of *Ccm2* in neonatal mice induces robust cerebellar CCMs [21]. Pregnant *iCre*;*Ccm2^fl/fl^* mice received tamoxifen by intragastric gavage at embryonic Day 18.5 (E18.5), and multiple CCM lesions were observed in the brains of their 14‐day‐old offspring (Figures 2A and S3A). Given that complete *Yap*/*Taz* double knockout causes lethal vascular defects [16], we investigated the impact of heterozygous *Yap* or *Taz* deficiency (*iCre*;*Ccm2^fl/fl^*;*Yap^fl/+^*, *iCre*;*Ccm2^fl/fl^*;*Taz^fl/+^*, and *iCre*;*Ccm2^fl/fl^*;*Yap^fl/+^*;*Taz^fl/+^*). Micro‐CT and histological analyses revealed that heterozygous deletion of *Yap* alone (*iCre*;*Ccm2^fl/fl^*;*Yap^fl/+^*) or in combination with *Taz* (*iCre*;*Ccm2^fl/fl^*;*Yap^fl/+^*;*Taz^fl/+^*) markedly reduced both lesion size and frequency (Figure 2A,B). Conversely, *Taz* deficiency alone failed to alleviate lesion burden (Figure S3A,B). Evans blue assays confirmed that vascular permeability remained intact across adult genotypes (Figure S3C,D); however, immunofluorescence staining showed that *Ccm2* loss compromised endothelial tight junctions (ZO‐1) and adherens junctions (VE‐cadherin) (Figure S4A,B). Crucially, deleting *Yap* or *Yap*/*Taz* restored the normal distribution of ZO‐1 and VE‐cadherin (Figure S4A,B). Furthermore, *Ccm2* deficiency‐induced upregulation of KLF4 in the cerebellum and aorta was substantially blunted by *Yap* or *Yap*/*Taz* knockout in vivo (Figures 2C and S5A,B) and in vitro (Figure 2D). Knockdown experiments in bEnd.3 cells confirmed that *Yap*, but not *Taz*, silencing diminished KLF4 expression (Figure S5C), establishing YAP as the primary upstream regulator of KLF4 in CCMs.

**FIGURE 2:**
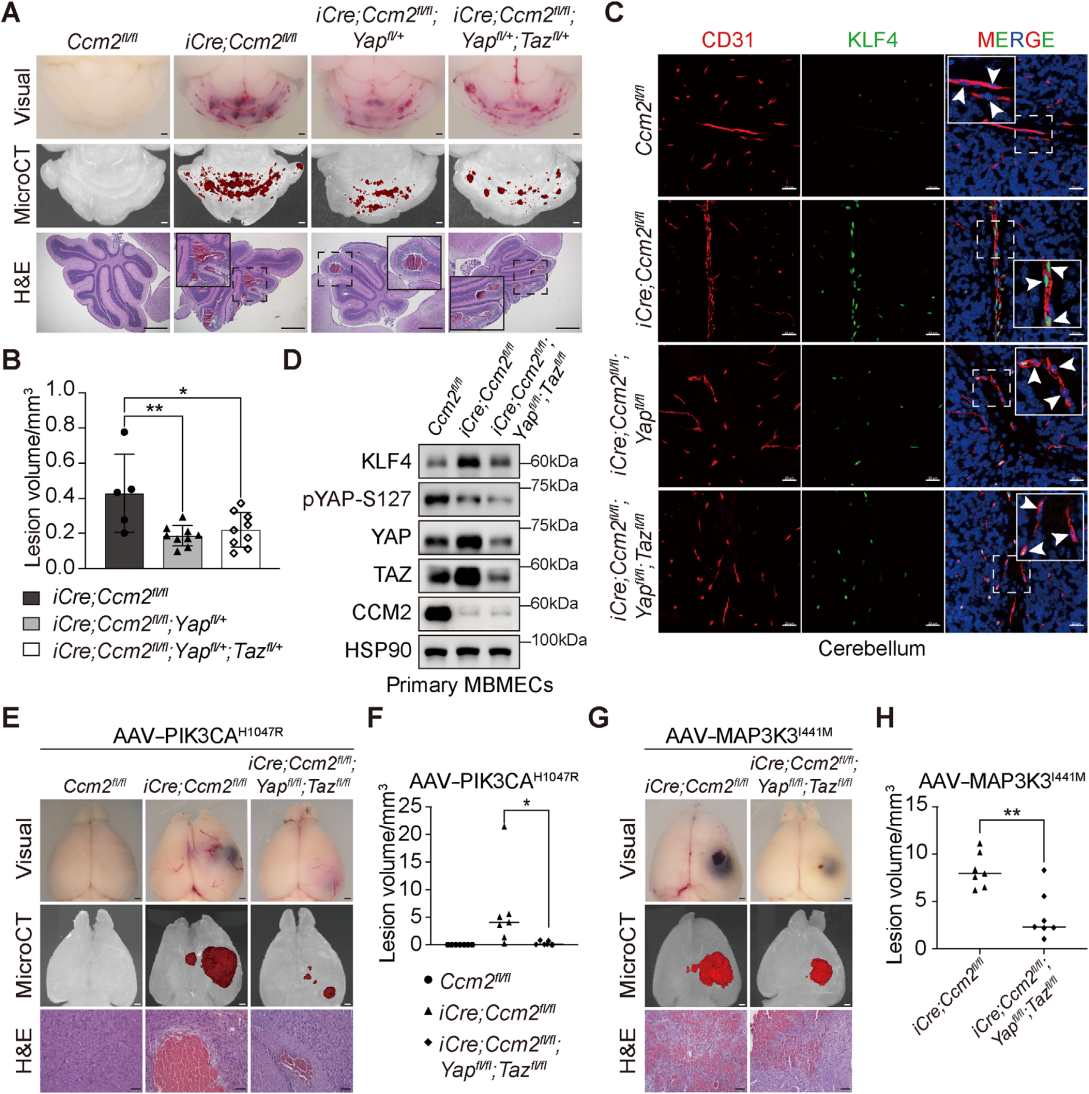
Endothelial deletion of *Yap* alleviates CCM progression. (A) Representative gross morphology (top), micro‐CT (medium), and H&E (bottom) images of mouse brains from the indicated genotypes at postnatal Day 14 (P14). Scale bars: 500 µm. (B) Quantification of P14 lesion volumes by micro‐CT analysis. *n* *= *5 mice for *Ccm2^fl/fl^*, *n* *= *5 mice for *iCre*;*Ccm2^fl/fl^*, *n* = 9 mice for *iCre*;*Ccm2^fl/fl^*;*Yap^fl/+^*, and *n* = 9 mice for *iCre*;*Ccm2^fl/fl^*;*Yap^fl/+^*;*Taz^fl/+^*. (C) Immunofluorescence staining of KLF4 (green) and CD31 (red) in the adult mouse cerebellum of the indicated genotypes. Nuclei were counterstained with DAPI (blue). White arrows indicate endothelial KLF4 signals in the cerebellar vessels. *n* = 3 mice per genotype. Scale bars: 20 µm. (D) Western blot analysis of KLF4 expression in primary MBMECs harvested from adult *Ccm2^fl/fl^, iCre*;*Ccm2^fl/fl^* and *iCre*;*Ccm2^fl/fl^*;*Yap^fl/fl^*;*Taz^fl/fl^* mice. (E) Representative gross morphology (top), micro‐CT (medium), and H&E (bottom) images of brains from adult *Ccm2^fl/fl^*, *iCre*;*Ccm2^fl/fl^* and *iCre*;*Ccm2^fl/fl^*;*Yap^fl/fl^*;*Taz^fl/fl^* mice collected 30 days after intracranial injection of AAV–BR1–PIK3CA^H1047R^. Scale bars: 1 mm (gross morphology and micro‐CT images); 50 µm (H&E images). (F) Quantification of lesion volumes from (E) by micro‐CT analysis (*n* = 7 per group). (G) Representative gross morphology (top), micro‐CT (medium), and H&E (bottom) images of brains from adult *iCre*;*Ccm2^fl/fl^* and *iCre*;*Ccm2^fl/fl^*;*Yap^fl/fl^*;*Taz^fl/fl^* mice collected 10 days after intracranial injection of MAP3K3^I441M^ AAV–BR1. Scale bars: 1 mm (gross morphology and micro‐CT images); 50 µm (H&E images). (H) Quantification of lesion volumes from (G) by micro‐CT analysis (*n* = 7 per group). Statistical analyses were performed using one‐way ANOVA followed by Dunnett's multiple comparison test (B) or unpaired Student's *t*‐test (F and H). Data are shown as mean ± SEM; *p < 0.05; **p < 0.01.

In addition to LOF mutations in the CCM complex, endothelial TLR4 activation and GOF mutations in *PIK3CA* and *MAP3K3* also contribute to CCM formation [5, 8, 22, 23]. In primary MBMECs, inflammatory stimulation with LPS or TNFα activated TLR signaling, decreased pLATS levels, and upregulated KLF4 and YAP protein levels (Figure S6A,B). LPS also induced YAP nuclear localization in bEnd.3 cells (Figure S6C). In *Ccm2*‐deficient primary MBMECs, LPS and TNFα stimulation further synergized to elevate KLF4, YAP, and TAZ protein levels (Figure S6D,E). Genetic ablation of *Yap* and *Taz* successfully blocked this inflammation‐induced KLF4 and *Adamts4/5* upregulation (Figure S6F–H). In vivo, *Yap* heterozygosity effectively protected neonatal *iCre*;*Ccm2^fl/fl^* mice from LPS‐exacerbated CCM lesion formation (Figure S6I). Collectively, endothelial *Yap* deletion alleviates CCM formation under inflammatory stimulation.

GOF mutations in *PIK3CA* (*e.g., PIK3CA^H1047R^*) are frequently found in human CCM lesions and act as an additional growth stimulus [9, 23]. Overexpression of PIK3CA and its active form PIK3CA^H1047R^ in bEnd.3 cells upregulated YAP protein levels (Figure S7A), whereas AKT inhibition via MK‐2206 reduced YAP protein levels in both WT and *Ccm2*‐deficient primary MBMECs (Figure S7B,C). To test the requirement of YAP/TAZ in PIK3CA GOF mutation‐driven CCM pathogenesis, we intracranially injected an AAV–BR1 virus carrying PIK3CA^H1047R^ into adult *Ccm2^fl/fl^, iCre*;*Ccm2^fl/fl^*, and *iCre*;*Ccm2^fl/fl^*;*Yap^fl/fl^*;*Taz^fl/fl^* mice. PIK3CA^H1047R^ accelerated lesion formation in *Ccm2*‐deficient adult mice but had no effect in *Ccm2^fl/fl^* controls (Figures 2E,F and S7D). Strikingly, *Yap* and *Taz* deletion markedly reduced lesion formation in the *Ccm2*‐deficient background (Figures 2E,F and S7D,E). Similarly, endothelial‐targeted delivery of the prevalent human CCM mutation MAP3K3^I441M^ via AAV–BR1 induced severe lesions in adult *Ccm2*‐deficient mice [8], which were significantly rescued by *Yap* and *Taz* deletion (Figures 2G,H and S7F,G). Taken together, these findings demonstrate that YAP/TAZ deficiency provides broad therapeutic protection against CCM pathogenesis driven by diverse genetic and inflammatory factors.

### 2.3 Endothelial Loss of LATS Kinase Upregulates KLF4 to Promotes CCM Progression

LATS1/2, the core kinase cascade of the Hippo pathway, directly phosphorylates YAP at Ser127, thereby inhibiting its nuclear localization and transcriptional activity [11, 24]. In line with reduced pYAP–S127 levels, LATS phosphorylation was markedly decreased upon *Ccm2* knockout, as well as following *Ccm1* or *Ccm3* deletion (Figures 1C and S1D,F,H,J). To investigate whether LATS kinase regulates KLF4 expression, we silenced *Lats1* in bEnd.3 cells, which provoked an upregulation of KLF4 and its downstream targets, parallel to increased YAP and TAZ levels (Figures 3A and S8A). In addition, we treated bEnd.3 cells with LATS‐IN‐1, an ATP‐competitive LATS1/2 kinase inhibitor effectively hindering LATS kinase‐mediated YAP phosphorylation [25]. Although the phosphorylation level of LATS increased after treatment, its activity was suppressed, as shown by reduced pYAP‐S127 and elevated expression of YAP target gene *Ctgf* and *Cyr61* (Figure S8B,C). Notably, LATS inhibition robustly increased KLF4 at both mRNA and protein levels (Figure S8B,C).

**FIGURE 3:**
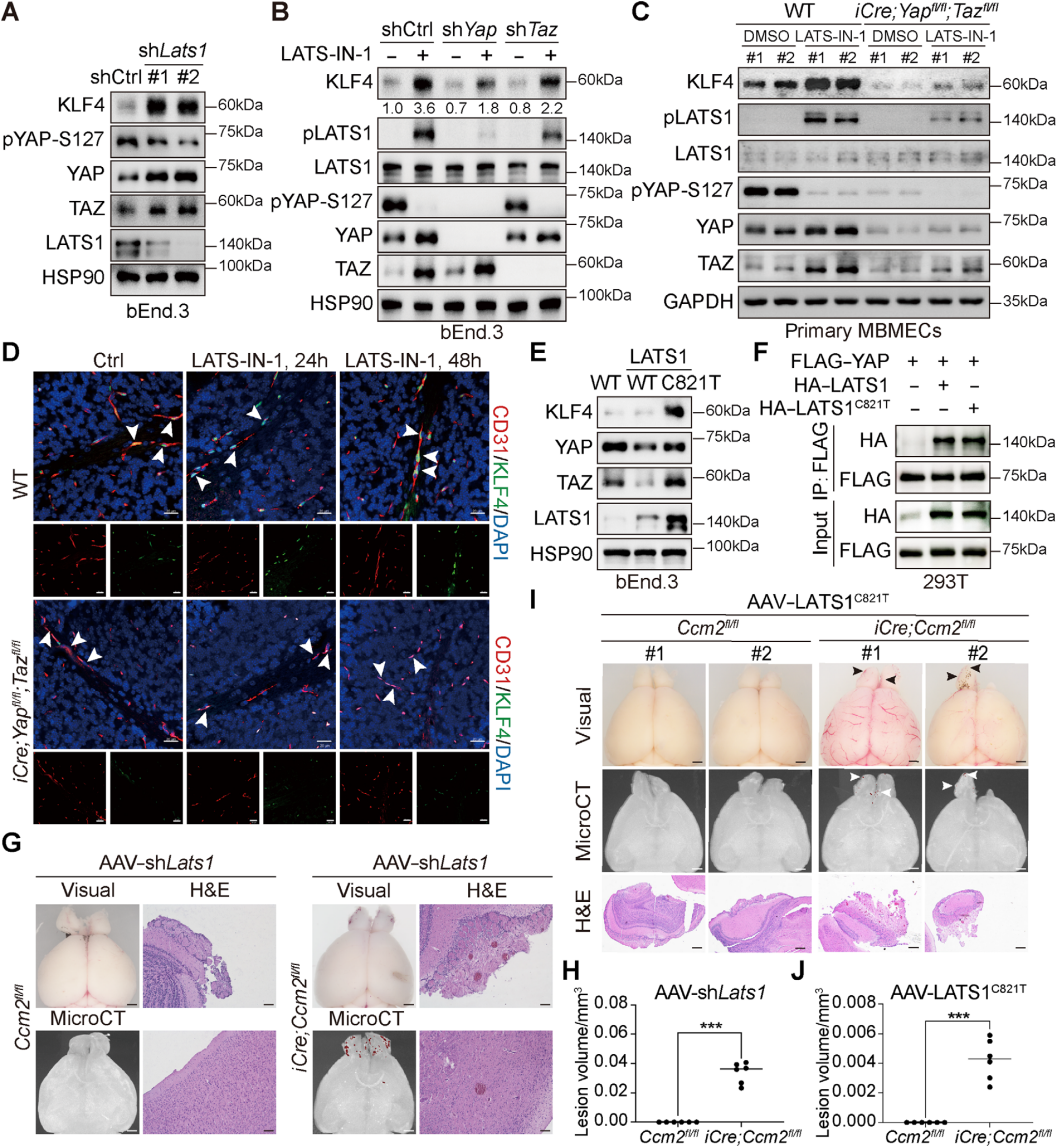
Endothelial loss of LATS kinase upregulates KLF4 and promotes CCM progression. (A) Western blot analysis of YAP and KLF4 protein levels in *Lats1*‐knockdown bEnd.3 cells. (B) Western blot analysis of KLF4 protein level in *Yap*‐knockdown bEnd.3 cells treated with the LATS kinase inhibitor LATS‐IN‐1 (10 µM, 24 h). (C) Western blot analysis of KLF4 protein levels in primary MBMECs isolated from adult *Yap^fl/fl^*;*Taz^fl/fl^* and *iCre*;*Yap^fl/fl^ Taz^fl/fl^* mice treated with LATS‐IN‐1 (10 µM, 24 h). (D) Immunofluorescence staining of KLF4 (green) and CD31 (red) in the adult cerebellum following tail vein injection of LATS‐IN‐1. Adult WT and *iCre*;*Yap^fl/fl^*;*Taz^fl/fl^* mice were intravenously injected with either vehicle or LATS‐IN‐1 (1 mg per injection in working solution) and sacrificed at 24 h (single injection) or 48 h (injections at 0 and 24 h). Nuclei were counterstained with DAPI (blue). Arrows indicate KLF4 signals in ECs. *n* = 3 mice per group. Scale bars: 20 µm. (E) Western blot analysis of YAP and KLF4 protein levels in bEnd.3 cells overexpressing LATS1 or LATS1^C821T^ mutant via lentiviral transduction. (F) Co‐IP analysis of the interaction between YAP and LATS1 or LATS1^C821T^. HEK293T cells were transiently cotransfected with FLAG–YAP and HA– LATS1 or HA–LATS1^C821T^ plasmids. FLAG–YAP proteins were immunoprecipitated, and HA‐tagged LATS1 proteins were detected by immunoblotting. (G) Representative gross morphology, micro‐CT, and H&E images of brains collected 60 days after injection of AAV–sh*Lats1* into adult *Ccm2^fl/fl^* and *iCre*;*Ccm2^fl/fl^* mice. Scale bars: 1 mm (gross morphology and micro‐CT images); 50 µm (H&E images). (H) Quantification of lesion volumes from (G) by micro‐CT analysis (*n* = 6 mice per group). (I) Representative gross morphology, micro‐CT, and H&E images of brains collected 60 days after injection of AAV–LATS1^C821T^ into adult *Ccm2^fl/fl^* and *iCre*;*Ccm2^fl/fl^* mice. Scale bars: 1 mm (gross morphology and micro‐CT images); 50 µm (H&E images). (J) Quantification of lesion volumes from (I) by micro‐CT analysis (*n* = 6 mice per group). Statistical analyses were performed using unpaired Student's *t*‐test (H and J). Data are shown as mean ± SEM; ***p < 0.001.

We next assessed whether LATS deficiency‐induced KLF4 upregulation depends on YAP/TAZ. Selective shRNA‐mediated knockdown of *Yap* or *Taz* in bEnd.3 cells revealed that *Yap* depletion, rather than *Taz* depletion, significantly blunted LATS‐IN‐1‐induced KLF4 upregulation (Figure 3B). Similarly, the induction of KLF4 by LATS‐IN‐1 observed in primary control MBMECs was almost entirely abolished upon combined *Yap* and *Taz* knockout (Figure 3C), establishing YAP as the predominant downstream mediator. To validate these findings in vivo, LATS‐IN‐1 was administered via tail vein injection. LATS‐IN‐1 administration robustly induced endothelial KLF4 expression within the cerebral and aortic vasculature of adult control mice, whereas this effect was severely attenuated in adult *iCre*;*Yap^fl/fl^*;*Taz^fl/fl^* mice (Figures 3D and S8D).

A recent clinical study identified a LATS1 missense mutation (c.821C>T, p.Thr274Ile) in CCM patients as a potential pathogenic variant [26]. However, its functional impact remains elusive. Overexpression of LATS1^C821T^ in bEnd.3 cells increased YAP and TAZ protein levels compared with WT LATS1 (Figure 3E). Co‐immunoprecipitation (Co‐IP) showed that while this mutation did not alter LATS1 binding affinity for YAP or TAZ, it effectively suppressed endogenous LATS activity, thereby classifying it as a LOF variant (Figures 3F and S8E). Notably, LATS1^C821T^ overexpression sufficed to upregulate KLF4 (Figure 3E), supporting its pathogenic relevance in CCM.

To evaluate the contribution of LATS1 inactivation to lesion formation, we intracranially injected an Adeno‐associated virus (AAV)–BR1 vector encoding *Lats1* shRNA into tamoxifen‐injected, disease‐sensitized adult *iCre*;*Ccm2^fl/fl^* mice. Although only a few small CCM lesions were observed at the injected sites, prominent hemorrhagic lesions consistently developed within the highly vascularized olfactory bulb (Figures 3G,H and S8F). Similarly, intracranial delivery of AAV–BR1 carrying LATS1^C821T^ into *iCre;Ccm2^fl/fl^* mice predominantly triggered hemorrhagic lesions in the olfactory bulb (Figure 3I,J). Intrigued by the temporal dynamics, we noted that lesion onset under LATS1 inhibition or mutation occurred around 2 months postinjection—markedly delayed compared with the 1‐month onset induced by the constitutively active YAP‐5SA mutant. Furthermore, the vascular phenotypes induced by *Lats1* shRNA or LATS1^C821T^ were generally milder than those elicited by YAP‐5SA. Successful transduction was validated by immunohistochemical staining of the AAV‐tagged proteins (Figure S8G,H). These spatial and temporal variations imply that YAP abundance, activity, and compensatory regulation by LATS1 are governed by sophisticated, multilevel control mechanisms during CCM pathogenesis.

### 2.4 YAP Physically Interacts With KLF4 to Cooperatively Drive Its Transcriptional Program

YAP has been shown to cooperate with KLF4 during intestinal epithelial differentiation [27]. To characterize this interaction in ECs, we performed Co‐IP assays in HEK293T cells and confirmed that YAP binds both KLF4 (Figure 4A) and KLF2 (Figure S9A). Similarly, TAZ interacted with both KLF4 and KLF2 (Figure S9B,C). Endogenous Co‐IP in bEnd.3 cells further validated the YAP–KLF4 complex formation, which was noticeably enhanced following *Ccm2* ablation (Figure 4B). To delineate the binding domain, we purified a recombinant KLF4 protein and its variant lacking repression domain (TRD) [28, 29]. In vitro pull‐down assays demonstrated that YAP directly binds to both full‐length KLF4 and its truncated variant (181–505 aa, spanning the transcriptional activation and C‐terminal zinc finger DNA‐binding domains) (Figure 4C). Consistently, proximity ligation assay (PLA) in primary MBMECs corroborated that endogenous YAP and KLF4 directly interact primarily within the nucleus, and this interaction was significantly enhanced by *Ccm2* deficiency (Figure 4D).

**FIGURE 4:**
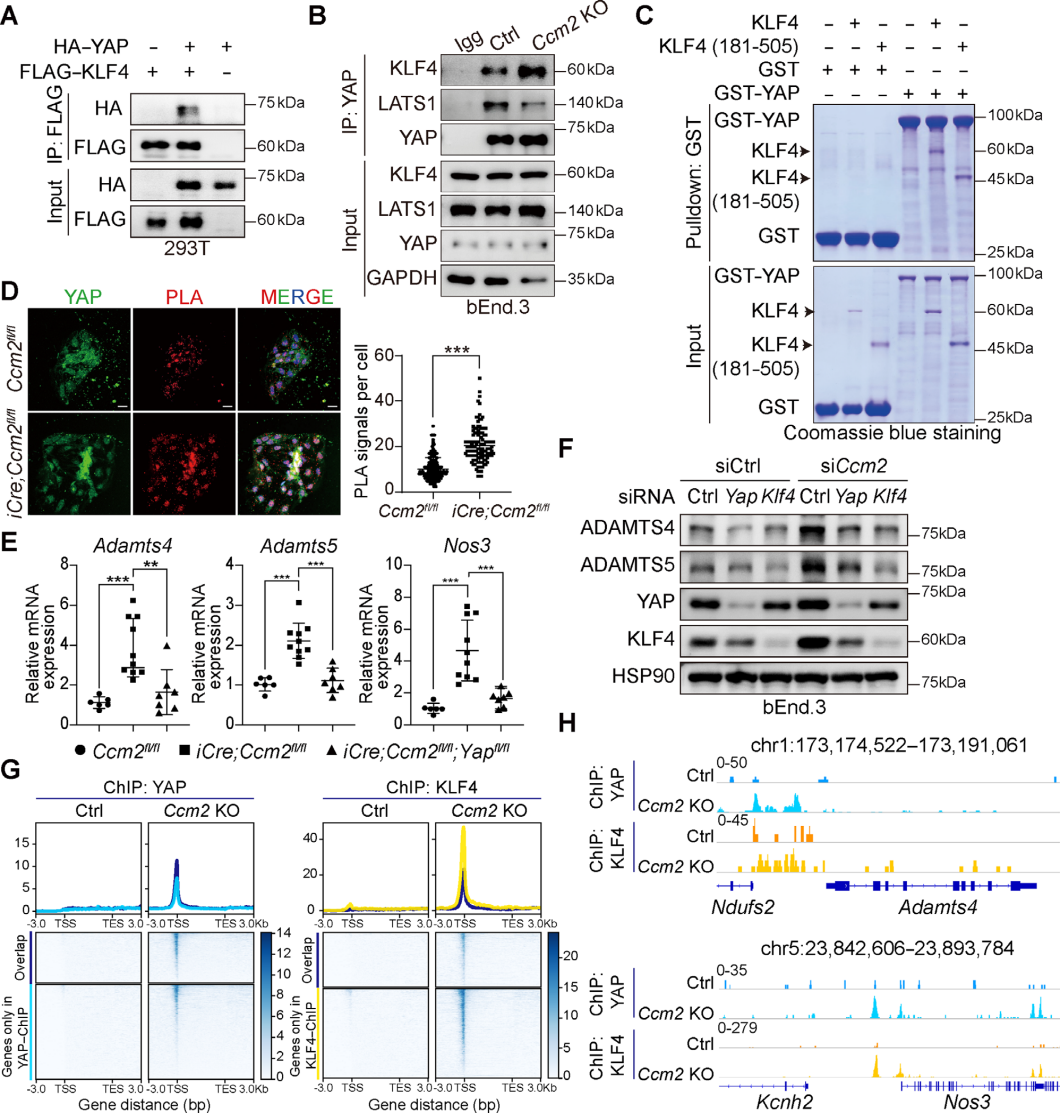
YAP interacts with KLF4 and promotes its transcriptional activity. (A) Co‐IP analysis of the interaction between YAP and KLF4. HEK293T cells were transiently cotransfected with FLAG–KLF4 and HA–YAP plasmids. FLAG–KLF4 was immunoprecipitated, and HA–YAP was detected by immunoblotting. (B) Endogenous Co‐IP analysis of YAP interactions with KLF4 and LATS1 in *Ccm2*‐knockout bEnd.3 cells. Endogenous YAP was immunoprecipitated, and immunoprecipitates were immunoblotted with anti‐KLF4 and anti‐LATS1 antibodies. (C) In vitro GST pull‐down assay of direct YAP–KLF4 binding. Recombinant GST–YAP, full‐length KLF4, and a truncated KLF4 mutant (181–505 aa) were purified from *E. coli* and subjected to GST pull‐down assays. Associated proteins were detected by Coomassie brilliant blue staining. (D) PLA analysis of endogenous YAP–KLF4 interaction in primary MBMECs harvested from adult *Ccm2^fl/fl^* and *iCre*;*Ccm2^fl/fl^* mice. Red fluorescent puncta indicate direct in situ interactions between YAP and KLF4. Quantification of PLA puncta per cell is shown in the right panel (*n* = 3 mice per genotype; >50 cells analyzed per mouse). (E) RT‐qPCR analysis of *Adamts4*, *Adamts5*, and *Nos3* mRNA levels in primary MBMECs isolated from adult mice of the indicated genotypes (*Ccm2^fl/fl^*, *n* = 6 mice; *iCre*;*Ccm2^fl/fl^*, *n* = 10 mice; *iCre*;*Ccm2^fl/fl^*;*Yap^fl/fl^*, *n* = 7 mice). (F) Western blot analysis of ADAMTS4 and ADAMTS5 protein levels in WT and *Ccm2*‐knockdown bEnd.3 cells with or without concurrent *Yap* or *Klf4* knockdown. (G) Average genomic profile and Heatmap of CUT&Tag sequencing datasets. Primary MBMECs isolated from adult *Ccm2^fl/fl^* and *iCre*;*Ccm2^fl/fl^* mice were subjected to CUT&Tag mapping using anti‐YAP or anti‐KLF4 antibodies to capture respective genome‐wide chromatin binding tracks. (H) Representative CUT&Tag genomic tracks visualizing YAP and KLF4 enrichment at the *Adamts4* and *Nos3* loci in primary MBMECs from *Ccm2^fl/fl^* and *iCre*;*Ccm2^fl/fl^* mice. Statistical analyses were performed using unpaired Student's *t*‐test in (D) or one‐way ANOVA followed by Dunnett's multiple comparison test in (E). Data are shown as mean ± SEM; **p < 0.01; ***p < 0.001.

To test whether YAP is required for KLF4‐mediated transcription, we evaluated the expression of *Adamts4/5* and *Nos3*. *Yap* genetic ablation or silencing significantly blunted the upregulation of *Adamts4/5* and *Nos3* in both primary MBMECs and bEnd.3 cells (Figures 4E,F and S9D–F). To globally map how YAP modulates KLF4 transcriptional activity, we performed RNA‐seq on bEnd.3 cells overexpressing YAP, KLF4, or both. Principal component analysis segregated control cells from all overexpression groups, with the YAP + KLF4 co‐overexpression cohort positioning intermediate to the distinct YAP–OE and KLF4–OE clusters (Figure S9G). Heatmap analysis revealed distinct expression patterns among groups (Figure S9H). Transcriptomic analysis identified 148 downregulated and 310 upregulated differentially expressed genes shared between the YAP–OE and KLF4–OE groups, underscoring a shared gene regulatory network (Figure S9I, J). Gene Ontology enrichment analysis revealed that codownregulated genes were heavily enriched in “regulation of angiogenesis” and “vasculature development” (Figure S9K), whereas coupregulated genes clustered in “cytokine‐mediated signaling” and “cell chemotaxis” (Figure S9L), highlighting a synergistic disruption of vascular homeostasis.

To define the genome‐wide chromatin dynamics, we performed Cleavage Under Targets & Tagmentation (CUT&Tag) sequencing in primary MBMECs isolated from *Ccm2^fl/fl^* and *iCre*;*Ccm2^fl/fl^* mice. Profiling of transcription start sites identified genes with either exclusive (YAP or KLF4 alone) or overlapping (both YAP and KLF4) enrichment. *Ccm2* deletion substantially expanded both the exclusive and overlapping target gene repertoires (Figure 4G). Genomic track visualization via the Integrative Genomics Viewer demonstrated that *Ccm2* loss remarkably amplified YAP‐binding peaks at the promoter regions of pathogenic genes *Adamts4*, *Nos3*, and TEAD target genes *Cyr61*, and *Ctgf* (Figures 4H and S9M,N). Strikingly, KLF4 CUT&Tag profiling revealed robust enrichment at the *Adamts4* and *Nos3* loci, whereas the *Cyr61* and *Ctgf* loci exhibited only modest enrichment (Figures 4H and S9N). Taken together, these findings demonstrate that CCM complex disruption unleashes a cooperative YAP–KLF4 transcriptional complex on chromatin, directly driving the expression of critical effectors that fuel CCM progression.

### 2.5 MAP3K3 Phosphorylates YAP to Promote Its Interaction With KLF4

We previous reported that MAP3K3 directly phosphorylates YAP at Ser371 and Thr412 [19], raising the possibility that MAP3K3 regulates YAP–KLF4 interaction via direct phosphorylation. Co‐IP assays in HEK293T cells showed that MAP3K3, but not its kinase‐dead mutant of MAP3K3 (MAP3K3‐KD), promoted the YAP–KLF4 interaction (Figure 5A). Similar results were observed for TAZ–KLF4 binding (Figure S10A).

**FIGURE 5:**
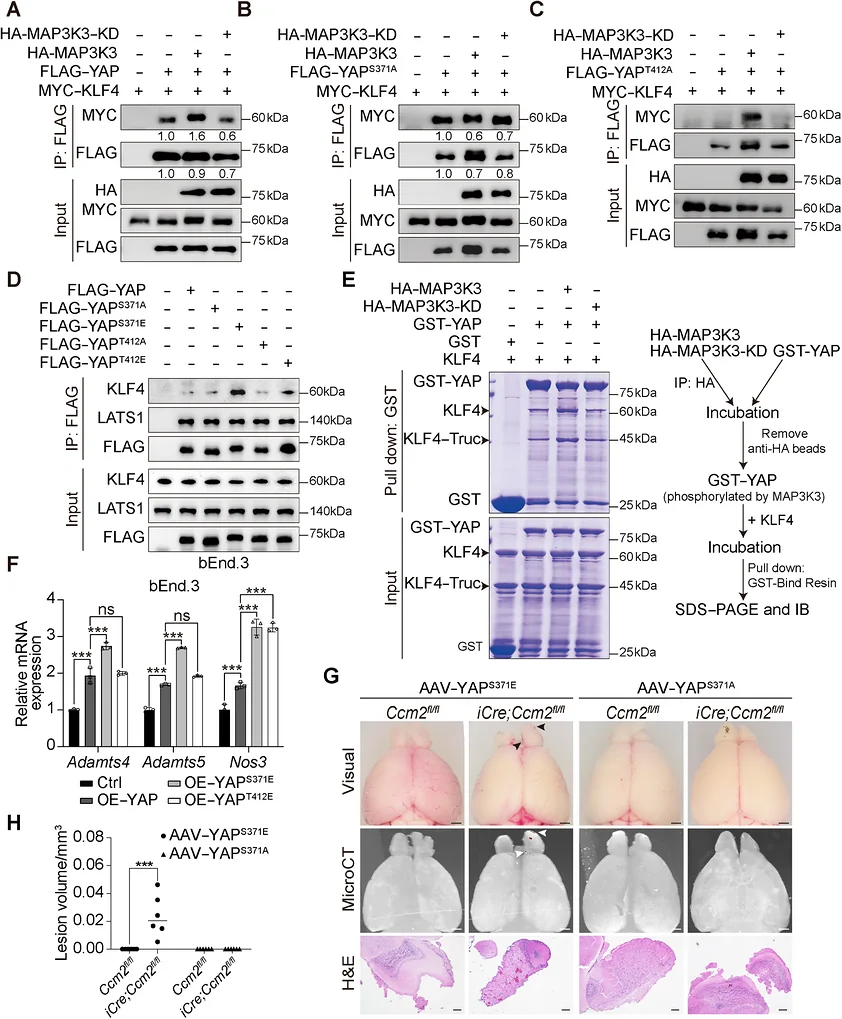
MAP3K3 phosphorylates YAP to promote its interaction with KLF4. (A–C) Co‐IP analysis of KLF4 binding to WT YAP (A), YAP^S371A^ (B), or YAP^T412A^ (C) in the presence of HA–MAP3K3 or its kinase‐dead mutant (HA–MAP3K3‐KD) in HEK293T cells. FLAG‐tagged YAP variants were immunoprecipitated, and copurified MYC–KLF4 was detected by immunoblotting. Numbers below the blots indicate relative binding intensity quantified by densitometry and normalized to the corresponding input control. (D) Co‐IP analysis of endogenous KLF4 and LATS1 binding to WT or mutant YAP in bEnd.3 cells. bEnd.3 cells were transduced with lentivirus expressing FLAG–YAP, FLAG–YAP^S371A^, FLAG–YAP^S371E^, FLAG–YAP^T412A^, or FLAG–YAP^T412E^. FLAG‐tagged proteins were immunoprecipitated, and endogenous KLF4 and LATS1 were detected by immunoblotting. (E) In vitro kinase/binding assay of YAP–KLF4 interaction. HA–MAP3K3 and HA–MAP3K3–KD were immunoprecipitated from HEK293T cells using anti‐HA magnetic beads and incubated with recombinant GST–YAP and KLF4 proteins purified from *E. coli*. The associated proteins were detected by Coomassie bright blue staining. The schematic diagram of the experimental workflow is shown in the right panel. (F) RT‐qPCR analysis of *Adamts4*, *Adamts5*, and *Nos3* mRNA levels in bEnd.3 cells expressing YAP^S371E^ or YAP^T412E^ mutants (*n* = 3 per group). (G) Representative gross morphology, micro‐CT, and H&E images of brains collected 30 days postintracranial injection of AAV expressing YAP^S371E^ or YAP^S371A^ into adult *Ccm2^fl/fl^* and *iCre*;*Ccm2^fl/fl^* mice. Arrows indicate hemorrhage sites. Scale bars: 1 mm (gross morphology and micro‐CT images); 50 µm (H&E images). (H) Quantification of lesion volumes from (G) by micro‐CT analysis (*n* = 6 mice per group). Statistical analyses were performed using two‐way ANOVA followed by Tukey's multiple comparisons test (F and H). Data are shown as mean ± SEM; n.s., not significant, *p* > 0.05; ***p < 0.001.

To decipher whether MAP3K3 regulates YAP–KLF4 binding through phosphorylation of YAP at Ser371 and Thr412, we individually mutated these two sites to alanine (A), which cannot be phosphorylated by MAP3K3. Notably, MAP3K3‐induced enhancement of the KLF4 interaction was completely abolished by the YAP^S371A^ mutation (Figure 5B), whereas YAP^T412A^ mutant remained responsive to MAP3K3 (Figure 5C), indicating that Ser371 of YAP is the primary functional site. Consistently, lentiviral overexpression of a phosphomimetic mutant YAP^S371E^ in bEnd.3 cells yielded a substantially stronger baseline affinity for KLF4 than WT YAP (Figure 5D). Furthermore, LPS and TNFα stimulation failed to enhance YAP–KLF4 interaction in *Map3k3* knockout cells (Figure S10B), establishing MAP3K3 as an obligate node coupling inflammation to the YAP–KLF4 complex. To confirm direct regulation, we performed an in vitro kinase assay with GST pull‐down (Figure 5E). Recombinant GST‐YAP pre‐incubated with immunoprecipitated WT MAP3K3, but not the KD mutant, exhibited enhanced binding to KLF4 (Figure 5E), providing biochemical evidence that MAP3K3 directly drives this interaction.

We next examined whether MAP3K3‐mediated modulation of the YAP–KLF4 complex alters KLF4 transcriptional activity. In bEnd.3 cells, YAP^S371E^ displayed a significantly higher potency in driving *Adamts4/5* and *Nos3* expression compared with WT YAP or YAP^T412E^ (Figure 5F). The S94A mutation in YAP abolishes its interaction with TEAD [30, 31]. We overexpressed YAP, YAP^S94A^ or YAP^S371E^ in bEnd.3 cells and found that the effects of YAP^S94A^ on the expressions of *Adamts4*/*5* and *Nos3* were no different from those of YAP and YAP^S371E^, suggesting that YAP‐mediated potentiation of KLF4 transcriptional activity operates independently on TEAD transcription factors (Figure S10C).

Given that LATS1 is key kinase regulating YAP activity, we interrogated the functional interplay between the MAP3K3 and LATS1 cascades. YAP^S371E^ or YAP^S371A^ mutant did not influence LATS1‐mediated YAP Ser127 phosphorylation (Figure S10D). While *Map3k3* silencing reduced YAP and KLF4 expression without altering pLATS1 levels (Figure S10E,F). Conversely, *Lats1* ablation increased YAP and KLF4 expression (Figure S10E). Intriguingly, concurrent *Map3k3* knockdown partially blunted the *Lats1* deficiency‐induced accumulation of YAP and KLF4, yet their levels remained elevated relative to *Map3k3* knockdown alone (Figure S10E). Similar results were observed in *Ccm2*‐deficient cells, where *Map3k3* knockdown reduced YAP and KLF4 expression but the levels remained elevated compared with *Map3k3* knockdown alone (Figure S10F). These findings indicate that CCM2 deficiency unleashes YAP activity via a dual‐tiered mechanism: activation of MAP3K3 to sustain YAP/KLF4 interaction, and concurrent inhibition of LATS1 to relieves its suppressive effect on YAP.

To establish the pathological relevance of YAP Ser371 phosphorylation in vivo, AAV–BR1 vectors carrying YAP^S371E^ or YAP^S371A^ intracranially injected into the adult *Ccm2^fl/fl^* and *iCre;Ccm2^fl/fl^* mice. Remarkably, the phosphomimetic YAP^S371E^ accelerated the formation of characteristic CCM lesions within the olfactory bulb of *Ccm2*‐deficient mice, whereas the non‐phosphorylatable YAP^S371A^ failed to do so (Figures 5G,H and S10G). Successful viral transduction across cohorts was confirmed via immunohistochemical staining of the tagged proteins encoded in AAV vectors (Figure S10H). Collectively, these findings underscore that MAP3K3‐mediated phosphorylation of YAP at Ser371 is a critical molecular driver of CCM pathogenesis.

### 2.6 YAP Protects KLF4 From β‐TrCP‐Mediated Proteasomal Degradation

Endothelial *Yap* deletion reduced KLF4 protein levels without significantly affecting its mRNA levels in primary MBMECs (Figure 6A,B), implicating YAP in the regulation of KLF4 protein stability. To test this hypothesis, we treated WT, *Yap*, and *Taz* knockdown bEnd.3 cells with either lysosome inhibitor chloroquine (CQ) or the proteasomal inhibitor MG132 to block protein degradation. While *Yap* knockdown decreased baseline KLF4 protein levels, MG132 treatment completely rescued KLF4 expression and restored it to control levels, whereas CQ treatment had no effect (Figures 6C and S11A). This proteasome‐dependent stabilization was recapitulated in *Yap/Taz* double‐knockout primary MBMECs and knockdown bEnd.3 lines (Figures 6D and S11B). Furthermore, MG132 treatment blunted the loss of KLF4 protein caused by *Yap/Taz* deficiency and synergistically augmented KLF4 levels in YAP‐ or TAZ‐overexpressing cells (Figure S11C).

**FIGURE 6:**
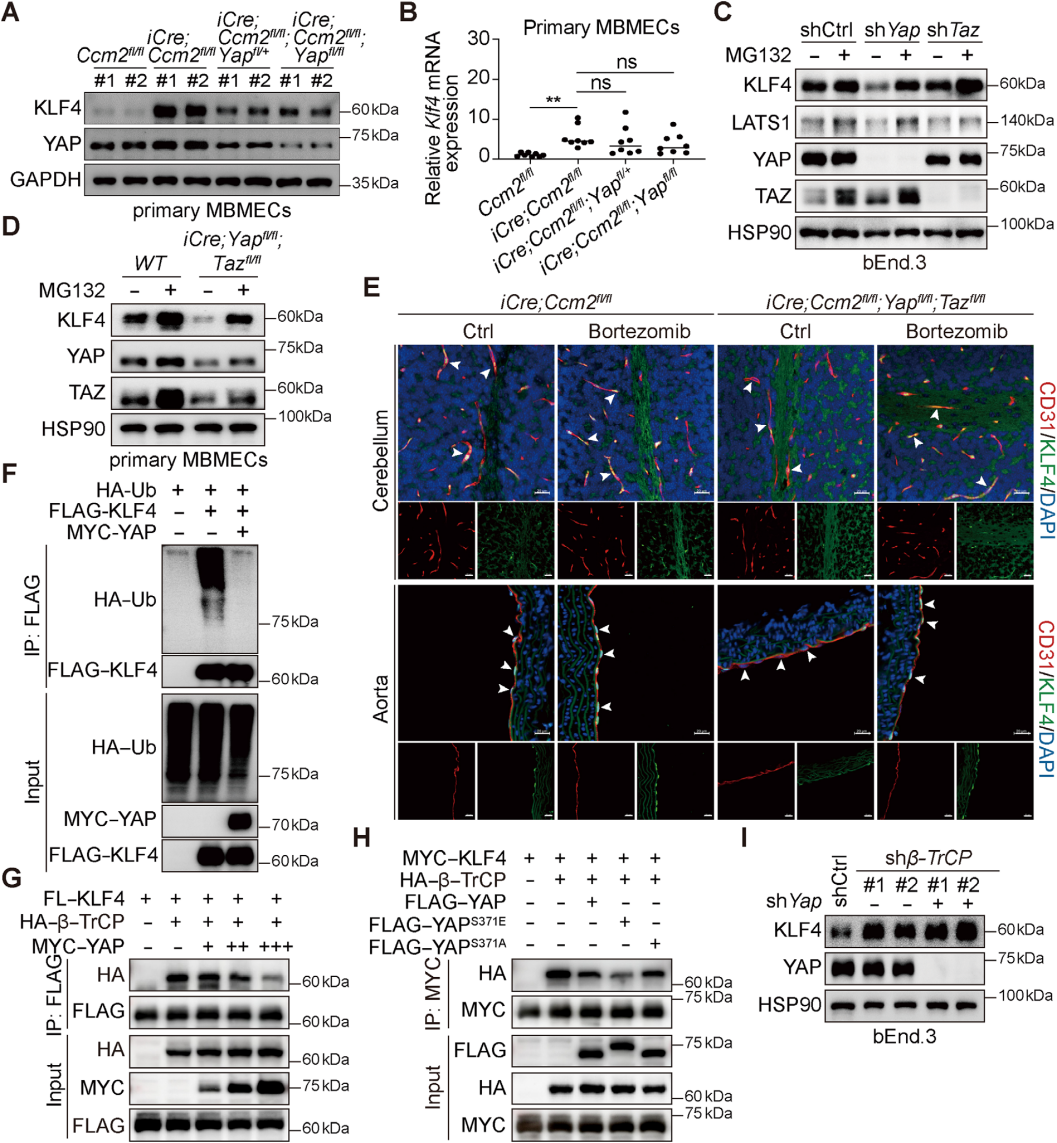
YAP protects KLF4 from β‐TrCP‐mediated proteasomal degradation. (A) Western blot analysis of KLF4 protein levels in primary MBMECs isolated from adult *Ccm2^fl/fl^, iCre*;*Ccm2^fl/fl^, iCre*;*Ccm2^fl/fl^*;*Yap^fl/+^*, and *iCre*;*Ccm2^fl/fl^*;*Yap^fl/fl^* mice. (B) RT‐qPCR analysis of *Klf4* mRNA levels in primary MBMECs from the indicated genotypes (*n* = 8 mice per group). (C) Western blot analysis of KLF4 protein levels in WT, *Yap*‐knockdown, and *Taz*‐knockdown bEnd.3 cells treated with or without MG132 (20 µM) for 4 h before harvest. (D) Western blot analysis of KLF4 protein levels in primary MBMECs harvested from adult WT and *iCre*;*Yap^fl/fl^*;*Taz^fl/f^* mice treated with MG132 (20 µM) for 4 h before harvest. (E) Immunofluorescence staining of KLF4 (green) and CD31 (red) in the cerebellum or aortic endothelium of adult *iCre*;*Ccm2^fl/fl^* and *iCre*;*Ccm2^flfl^*;*Yap^fl/fl^*;*Taz^fl/fl^* mice. Mice received three intraperitoneal injections of 100 µg tamoxifen followed by two intravenous injections of 20 µg bortezomib or vehicle within 24 h. Nuclei were counterstained with DAPI (blue). *n* = 3 mice per genotype. Scale bars: 20 µm. (F) Co‐IP analysis showing that YAP reduces ubiquitination of KLF4. HEK293T cells were transiently cotransfected with HA–Ub, FLAG–KLF4, with or without MYC–YAP plasmids. FLAG–KLF4 proteins were immunoprecipitated, and HA–Ub‐modified FLAG–KLF4 proteins were detected by immunoblotting. (G) Co‐IP analysis showing that YAP inhibits KLF4–β‐TrCP interaction in a dose‐dependent manner. HEK293T cells were transiently cotransfected with FLAG–KLF4, HA–β‐TrCP, and increasing amounts of MYC–YAP (0.3, 0.6, 0.9 µg). FLAG–KLF4 was immunoprecipitated, and HA–β‐TrCP was detected by immunoblotting. (H) Co‐IP analysis showing that the YAP^S371E^ phosphomimetic mutant displays an enhanced efficiency in disrupting the KLF4–β‐TrCP binding compared with WT YAP. HEK293T cells were transiently cotransfected with FLAG–KLF4, HA–β‐TrCP, and MYC–YAP or MYC–YAP^S371E^. FLAG–KLF4 was immunoprecipitated, and HA–β‐TrCP was detected by immunoblotting. (I) Western blot analysis of KLF4 protein levels in bEnd.3 cells transduced with sh*β‐TrCP* alone or together with sh*Yap* lentivirus. Statistical analyses were performed using one‐way ANOVA followed by Dunnett's multiple comparison test (B). Data are shown as mean ± SEM; n.s., not significant, *p* > 0.05; **p < 0.01.

This posttranslational regulation was further validated using bortezomib (BTZ), a clinically approved proteasome inhibitor, which mirrored the stabilizing effects of MG132 in vitro (Figure S11D). To corroborate these mechanisms in vivo, BTZ was administered to adult mice via tail vein injection. BTZ treatment substantially augmented endothelial KLF4 expression within the brain and aortic vasculature of control and *iCre*;*Ccm2^fl/fl^* mice (Figures 6E and S11E). Importantly, systemic proteasome inhibition by BTZ administration restored endothelial KLF4 protein levels in both *iCre*;*Yap^fl/fl^*;*Taz^fl/fl^* mice (Figure S11E) and *iCre*;*Ccm2^fl/fl^*;*Yap^fl/fl^*;*Taz^fl/fl^* mice (Figure 6E), reinforcing the notion that YAP controls KLF4 abundance primarily by gating its proteasomal degradation.

To elucidate the mechanism by which YAP regulates KLF4 degradation, we first assessed KLF4 ubiquitination upon YAP modulation. YAP overexpression profoundly suppressed KLF4 ubiquitination in HEK293T cells (Figure 6F). The E3 ligase β‐TrCP is known to catalyze KLF4 ubiquitination and to promote its degradation [32]. We thus reasoned that YAP might shield KLF4 from β‐TrCP‐mediated proteasomal degradation. Coexpression assays revealed that YAP overexpression displaced KLF4 from the β‐TrCP complex in a dose‐dependent manner, progressively destabilizing the β‐TrCP–KLF4 interaction (Figures 6G and S11F). Strikingly, the pathologically active YAP^S371E^ mutant was more effective at disrupting the β‐TrCP–KLF4 association than WT YAP (Figure 6H). To further establish the functional role of β‐TrCP, we silenced *β‐TrCP* in bEnd.3 cells (Figure S11G). Depletion of *β‐TrCP* alone induced a robust accumulation of KLF4, and crucially, *Yap* knockdown failed to destabilize KLF4 protein expression in the absence of *β‐TrCP* (Figure 6I). Taken together, these data demonstrate that activated YAP physically occupies or sterically hinders the KLF4 complex, protecting it from β‐TrCP‐mediated proteasomal degradation to sustain the pathogenic endothelial program in CCM.

## 3 Discussion

In this study, we uncover a previously unrecognized pathogenic role for the Hippo–YAP signaling cascade in CCMs, expanding our understanding of vascular lesion progression beyond the canonical MAP3K3–KLF2/4 axis. Mechanistically, our data support a model wherein CCM gene complex deficiency dual‐regulates YAP activity through a “dual‐pronged” molecular mechanism. First, it relieves LATS‐mediated negative suppression, thereby licensing YAP nuclear translocation. Second, it provokes MAP3K3‐mediated direct phosphorylation of YAP at Ser371, which structurally stabilizes its physical association with KLF4. Crucially, this robust interaction shields KLF4 from β‐TrCP‐mediated ubiquitination and subsequent proteasomal degradation in ECs, creating a pathogenic feed‐forward loop that accelerates vascular malformation.

The Hippo–YAP signaling cascade is a critical gatekeeper of cellular homeostasis and vascular integrity [11, 33]. Here, we uncover a novel regulatory role for LATS kinase and YAP in the pathogenesis of CCMs. Endothelial loss of *Ccm2* suppresses Hippo signaling, while LATS inhibition or expression of the clinical LATS1^C821T^ LOF variant markedly upregulates YAP/TAZ and KLF4, driving intracranial hemorrhage in mice. This establishes the LATS–YAP axis as a key pathogenic contributor to vascular malformations, extending our understanding beyond the canonical CCM pathways.

Current paradigms of CCM pathogenesis rely on genomic complexity, varying from the “two‐hit” or “three‐hit” hypotheses involving CCM loss and PIK3CA GOF mutations [9, 23], to the recently proposed “one‐hit” model driven by MAP3K3^I441M^ [6, 7]. While PIK3CA^H1047R^ has been reported to enhance YAP/TAZ activity [34], our study demonstrates that endothelial ablation of *Yap/Taz* effectively mitigates CCM lesions induced by either *Ccm2* deficiency or PIK3CA^H1047R^ expression, suggesting a crucial convergence point or bidirectional crosstalk between PI3K and YAP signaling. More importantly, we identified a previously unrecognized mechanism where MAP3K3 directly phosphorylates YAP, facilitating a highly stable YAP–KLF4 transcriptional interactome. Crucially, genetic ablation of *Yap* successfully alleviated MAP3K3^I441M^‐driven CCM progression, validating that YAP acts as a critical downstream effector across diverse genetic triggers.

Beyond specific genetic mutations, microenvironmental inputs like inflammation and hemodynamic shear stress are potent drivers of CCM disease [2, 35]. Although global YAP upregulation is commonly linked to inflammatory diseases [36], its complete endothelial deletion can paradoxically trigger vascular activation [37]. Our study highlights the necessity of an intricate homeostatic balance, showing that heterozygous loss of *Yap* significantly ameliorates CCM formation triggered by *Ccm2* deficiency or inflammatory stimuli (LPS/TNFα). Furthermore, given that KLF2/4 and YAP are individually recognized as mechanosensitive factors responsive to blood flow [38, 39, 40], the MAP3K3–YAP–KLF4 axis uncovered here likely serves as a critical signaling hub integrating hemodynamic forces during lesion expansion. Notably, unlike the steady laminar shear stress in healthy vessels, disturbed and recirculating blood flow within cavernous lesions may persistently retrigger the MAP3K3–YAP–KLF4 signaling axis in ECs, thereby establishing a self‐sustaining positive feedback loop that drives continuous lesion expansion.

While CCM2 acts primarily as a scaffold for MAP3K3 and CCM3 operates within the STRIPAK complex to potentially modulate MST1/2 [41, 42], our data confirm that both deficiencies converge on YAP activation. However, the architectural divergence in their respective Hippo regulatory mechanisms warrants future investigation. Nevertheless, the critical role of our proposed LATS/MAP3K3–YAP–KLF4 axis in CCM pathogenesis is tempered by several limitations. First, while our in vitro mechanistic data demonstrate that the posttranslational stabilization of KLF4 is explicitly mediated by its physical interaction with YAP, our in vivo validation relies on the systemic administration of small‐molecule inhibitors (LATS‐IN‐1 and BTZ). Given that systemic proteasome exerts broad, pleiotropic effects on vascular hemodynamics and microvascular inflammation (such as through the NF‐κB pathway), the resulting in vivo phenotypes cannot be exclusively ascribed to the endothelial YAP–KLF4 interface. Future studies utilizing endothelial‐specific, inducible knockout mice for individual phosphorylation site mutants will be necessary to decouple these systemic confounding factors. Second, while we identified a dual‐mechanism model in ECs wherein CCM2 deficiency simultaneously activates MAP3K3 and suppresses LATS1 to optimize YAP–KLF4 cooperative transcription, this net positive output contrasts with the LATS‐dependent inhibitory feedback loop previously reported in other cellular systems [19]. This discrepancy is highly likely due to the cell‐type‐specific context, wherein the baseline expression profiles and abundance of these core signaling components vary fundamentally across different biological settings. The exact molecular intermediaries dictating how PI3K GOF or CCM complex disruption fine‐tunes the biochemical crosstalk between the parallel MAP3K3 and LATS arms remain to be fully mapped. In addition, because some AAV experiments did not include empty/shCtrl AAV controls of the same genotype or sham controls, the potential confounding effects of injection, viral vectors, and background genotype cannot be fully excluded.

In conclusion, our study establishes the LATS/MAP3K3–YAP–KLF4 signaling axis as a foundational pillar of CCM pathogenesis. Targeting this axis presents a compelling therapeutic opportunity for CCM. However, systemic inhibition of broad‐spectrum targets like LATS kinases or the proteasome carries substantial risk of off‐target toxicity. Strategically disrupting the specific physical interaction interface between YAP and KLF4‐ or utilizing endothelial‐targeted delivery vehicles such as lipid nanoparticles or nanomedicines could offer a far safer, cell‐type‐specific intervention, effectively circumventing systemic adverse effects while selectively mitigating cavernous lesion progression

## 4 Methods

### 4.1 Human Brain Samples

Total 30 human CCM tissue specimens were obtained from the Vascular Surgery Clinical Research Database at the First Affiliated Hospital, School of Medicine, Zhejiang University. Control brain tissues were collected from 20 patients, including peritumoral normal brain tissues from CCM patients (*n* = 5), glioma tissues (*n* = 10), and meningioma tissues (*n* = 5). Paraffin‐embedded sections (6‐µm thick) were dewaxed and rehydrated through xylene and ethanol treatment, and used for immunofluorescence analysis. YAP expression in vascular ECs was quantified from 10 randomly selected fields of view per section under confocal microscopy.

### 4.2 Mice

*Cdh5^CreER^* mice were provided from Dr. Luyang Yu at Zhejiang University. *Ccm2^fl/fl^* mice were provided by Dr. Xiangjian Zheng at Tianjin Medical University. *Yap^fl/fl^* mice were provided by Dr. Pan Duojia, and *Taz^fl/fl^* mice were provided by Dr. Nan Tang at the National Institute of Biological Sciences. The indicated genotypes were generated by mating between these mice. The mice were maintained in a mixed genetic background and housed in specific pathogen‐free facility maintained at 25 ± 2°C and 12 h light/dark cycle. No statistical method was used to predetermine the gender and sample size in the animal studies.

For neonatal experiments, pregnant mice were intragastrically administered 100 µg tamoxifen (Sigma–Aldrich) dissolved in corn oil at E18.5 unless otherwise indicated. Pups were euthanized on day P14 to harvest their brains.

For adult experiments, mice at 4–6 weeks were injected intraperitoneally with 100 µg of tamoxifen dissolved in corn oil every other day three times unless otherwise indicated. For the isolation of primary MBMECs, the mice received five injections of tamoxifen to ensure a complete gene knockout.

For euthanasia and anesthesia, pentobarbital sodium was administered via intraperitoneal injection at doses of 200 and 50 mg/kg body weight, respectively. To ensure a humane endpoint, mice were placed in a chamber with a gradually increasing CO_2_ concentration to induce unconsciousness within 5 min. Death was confirmed by the absence of a heartbeat and reflex.

### 4.3 CRISPR/Cas9‐Mediated Genomic Editing

Guide RNA sequences targeting mouse *Ccm1*, *Ccm2*, *Ccm3*, and *Map3k3* were cloned into the plasmid PX459 (Addgene; #62988). shRNA sequences targeting mouse *Lats1*, *Yap1*, *Taz*, *Klf4*, and *β‐TrCP* were cloned into plasmid pLV3‐H1‐puro. siRNAs directed against mouse *Ccm2*, mouse *Map3k3*, and human *CCM2* were purchased from Invitrogen. Sequences of guide RNA, shRNA, and siRNA are listed in Table S1.

### 4.4 Transfection and Lentiviral Infection

For transfection, *Lats1*, *Yap1*, *Taz*, *Klf4, Map3k3*, and *β‐TrCP* cDNAs were cloned into pcDNA3.1. These constructs were subsequently transfected into HEK293T cells using polyethylenimine. For lentiviral infection, *Lats1*, *Yap1*, *Taz*, *Klf4, Map3k3*, and *β‐TrCP* cDNAs were cloned into pCDH‐CMV‐puro. Lentiviruses were generated in HEK293T cells with indicated plasmids and packaging plasmids pMD2.G (Addgene; #12259) and psPAX2 (Addgene; #12260). Viruses were harvested 48 and 72 h after transfection, and then incubated with bEnd.3 cells for 24 h in the presence of polybrene. The transduced cells were selected by puromycin with a final concentration of 1 µg/mL.

### 4.5 Primary MBMECs Harvesting

Adult mice received five intraperitoneal injections of tamoxifen every other day to induce endothelial‐specific gene knockout. Primary MBMECs were subsequently isolated from the whole brain according to previously reported protocols [43]. Briefly, the brain tissues were shredded with blades and centrifuged with 15% dextran (in sterile phosphate‐buffered saline [PBS]). The pellet was resuspended with serum‐free Dulbecco's modified eagle medium (DMEM) (Gibco) containing 10 µg/mL DNase I (Sangon Biotech) and 1% Type I collagenase (Worthington) and digested for 1 h at 37°C with gentle shaking. Subsequently, cells were centrifuged, resuspended in complete DMEM medium (10% fetal bovine serum, 1× penicillin/streptomycin), and cultured in 12‐well plates pretreated with 1% gelatin at 37°C and 5% CO_2_ for further analysis.

### 4.6 AAV Injection

Mice were administered three injections of tamoxifen to induce endothelial‐specific gene knockout. AAV–BR1 viruses carrying YAP‐5SA–FLAG, LATS1^C821T^–FLAG, YAP^S371E^‐FLAG, YAP^S371A^–FLAG, MAP3K3^I441M^–FLAG, or PIK3CA^H1047R^–HA under the control of CAG promoter, and AAV–BR1 carrying sh*Lats1*–mcherry–FLAG or shCtrl–mcherry under the control of TIE1p promoter, were injected into the right lateral hemisphere of the mice using microliter syringes. Total 1 × 10^10^ virus particles suspended in sterile PBS were injected over 5 min.

### 4.7 X‐Ray Micro‐CT Based Quantification of CCM Lesions

Mice were anesthetized with pentobarbital and subjected to intracardiac perfusion with PBS. Brains were dissected and fixed in 4% paraformaldehyde (PFA)/PBS overnight at 4°C. Subsequently, brains were rinsed several times with PBS, soaked in Lugol's iodine solution for 3 days, and scanned using a Micro‐CT system at 50 kV and 10 W. Raw image stacks were analyzed using MILabs Rec 10.16 software, and volumes of labeled lesions were quantified using IMALYTICS Preclinical 2.1 software.

### 4.8 Histology

Tissue samples were fixed in 4% PFA/PBS overnight at 4°C, dehydrated in 100% ethanol, subjected to xylene treatment, and embedded in paraffin. Sections (7‐µm thick) underwent dewaxing and rehydration through xylene and ethanol treatment and were used for immunofluorescence and H&E staining. Sections were stained with hematoxylin (Biosharp) for 1 min before rinsing with tap water for 5 min. Subsequently, sections were washed with 80% alcohol for 2 min and stained with eosin (Servicebio) for 3 s. After gradual dehydration, the sections were mounted with neutral balsam.

### 4.9 Immunofluorescence Staining

Sections were heated in boiling EDTA Antigen Retrieval Solution (pH 9) (Sangon Biotech) and endogenous peroxidase activity was inhibited using 3% H_2_O_2_. After blocking with 5% bovine serum albumin (BSA) in PBST (0.1% Tween‐20), sections were incubated overnight with the following primary antibodies for immunostaining: rabbit anti‐KLF4, rabbit anti‐YAP, mouse anti‐ZO‐1, and rabbit anti‐VE‐cadherin. Biotinylated secondary antibody and horseradish peroxidase (HRP)‐conjugated streptavidin (1:1000; Jackson ImmunoResearch Laboratories) were used in combination with FITC‐tyramide (1:3000; TSA kit; Servicebio) to amplify the signals. The bound antibody was removed via heating to boiling in a citrate antigen retrieval solution (Sangon Biotech). After cooling to room temperature, the sections were washed with PBS and blocked with 5% BSA before incubation with rabbit anti‐CD31 antibody. Finally, the sections were incubated with Alexa Fluor 594 goat anti‐rabbit antibody (Hangzhou Baoke Biotechnology Co., Ltd.) and counterstained with 4′,6‐diamidino‐2‐phenylindole (DAPI). Antibody information is listed in Table S2.

### 4.10 Co‐Immunoprecipitation

After transfection with indicated plasmids for 24–36 h, HEK293T cells were collected in ice‐cold lysis buffer (50 mM Tris 7.4, 150 mM NaCl, 10% glycerin, and 1% NP‐40) containing 0.5 mM dithiothreitol (DTT), 1 mM phenylmethylsulfonyl fluoride (PMSF), protease, and phosphatase inhibitors, and then centrifuged at 15,000×*g* for 10 min at 4°C. Anti‐FLAG or anti‐HA magnetic beads (Thermo Scientific) were added to the supernatant, and the mixtures were incubated at 4°C for 6 h under gentle agitation. After washing three times with lysis buffer containing 0.5 mM DTT, 1 mM PMSF, protease, and phosphatase inhibitors, the beads were resuspended in 2×SDS loading buffer, and the eluted proteins were detected by immunoblotting.

### 4.11 PLA

Primary MBMECs from *Ccm2^fl/fl^* and *iCre;Ccm2^fl/fl^* mice were plated on coverslips and fixed with 4% PFA on ice for 20 min, followed by permeabilization with 0.5% Triton X‐100 (in PBS) for 5 min. Subsequently, the cells were blocked with 3% BSA/PBST for 1 h, and incubated with mouse anti‐YAP (1:100) and rabbit anti‐KLF4 (1:100) antibodies for 3 h at room temperature. Then, antibody labeling, ligation, and amplification were performed using a PLA kit (Sigma) according to the manufacturer's instructions. The direct interaction between YAP and KLF4 was visualized as red fluorescent dots.

### 4.12 Tail Vein Injection of Inhibitors

LATS‐IN‐1 (TargetMol) was dissolved in dimethyl sulfoxide (DMSO) to generate an 80 mg/mL stock solution and further diluted in normal saline containing 30% PEG300 (Sigma) to achieve a working solution of 10 mg/mL. Adult mice were injected intraperitoneally with tamoxifen or corn oil (serving as the control group) every other day for three times. Then, the mice were injected intravenously with either 100 µL LATS‐IN‐1 working solution or vehicle. The mice were injected twice within 48 h, and the brains and aortas were harvested.

BTZ was dissolved in DMSO to generate a 40 mg/mL stock solution, which was subsequently diluted in normal saline containing 40% PEG300 and 5% Tween‐80 to produce a working solution of 0.2 mg/mL. The mice were injected intravenously with either 100 µL BTZ working solution or vehicle. The mice were injected twice within 24 h, and the brains and aortas were harvested.

### 4.13 Statistical Analysis

All the experiments were performed in triplicates, and data are presented as mean ± standard errors of the means (SEM). Statistical analysis was performed using GraphPad Prism 10. Normality was assessed using the Shapiro–Wilk test, and homogeneity of variance was evaluated using Levene's test. Outliers were identified using the interquartile range method. For normally distributed data with one variable, differences between the two groups were analyzed using unpaired Student's *t*‐test, and differences among more than two groups were analyzed using one‐way ANOVA followed by Dunnett's multiple comparison test. For normally distributed data with two variables, differences were evaluated by two‐way ANOVA with Tukey's multiple comparisons test. Results with a *p* < 0.05 were considered statistically significant.

## Author Contributions

Hai Song, Kai Wang, and Tingting Ye: conceived the conception and experimental design; Tingting Ye and Zonghao Hu: carried out most of the experiments; Qi Zheng and Chengdong Chang: performed human CCM staining study; Haomin Mo: generated CRISPR/Cas9 vector; Haonan Fan and Zhongxing Sun: performed RNA‐seq and CUT&Tag data analysis; Xiya Li and Yanhua Xu: helped to perform animal study; Kai Wang and Dong Fang: contributed to intellectual inputs; Hai Song And Tingting Ye: wrote the manuscript. All authors have read and approved the final manuscript

## Conflicts of Interest

The authors declare no conflicts of interest.

## Ethics Statement

All animal experiments were approved by the Institutional Animal Care and Use Committee of Zhejiang University (ZJU20240078) and conducted in compliance with the NIH Guide for the Care and Use of Laboratory Animals. The human study was conducted in accordance with the ethical principles of the Declaration of Helsinki and was approved by the Clinical Research Ethics Committee of the First Affiliated Hospital, Zhejiang University School of Medicine (IIT20240669B). All patients provided written informed consent prior to participation.

## Supporting information




**Supporting Information file 1**: mco271086‐sup‐0001‐SuppMat.pdf

## Data Availability

Raw RNA‐seq data have been deposited in the GEO database under accession number GSE280200 and CUT&Tag data under GSE280201. All other data supporting the findings of this study are available within the article and its Supporting Information files.

## References

[1] E. R. Smith , “Cavernous Malformations of the Central Nervous System,” New England Journal of Medicine 390, no. 11 (2024): 1022–1028. 10.1056/NEJMra2305116 38477989

[2] D. A. Snellings , C. C. Hong , A. A. Ren , et al., “Cerebral Cavernous Malformation: From Mechanism to Therapy,” Circulation Research 129, no. 1 (2021): 195–215. 10.1161/CIRCRESAHA.121.318174 PMC8922476 34166073

[3] A. Fischer , J. Zalvide , E. Faurobert , C. Albiges‐Rizo , and E. Tournier‐Lasserve , “Cerebral Cavernous Malformations: From CCM Genes to Endothelial Cell Homeostasis,” Trends in Molecular Medicine 19, no. 5 (2013): 302–308. 10.1016/j.molmed.2013.02.004 23506982

[4] Z. Zhou , A. T. Tang , W. Y. Wong , et al., “Cerebral Cavernous Malformations Arise From Endothelial Gain of MEKK3–KLF2/4 Signalling,” Nature 532, no. 7597 (2016): 122–126. 10.1038/nature17178 PMC4864035 27027284

[5] A. T. Tang , J. P. Choi , J. J. Kotzin , et al., “Endothelial TLR4 and the Microbiome Drive Cerebral Cavernous Malformations,” Nature 545, no. 7654 (2017): 305–310. 10.1038/nature22075 PMC5757866 28489816

[6] J. Ren , Y. Huang , Y. Ren , et al., “Somatic Variants of MAP3K3 Are Sufficient to Cause Cerebral and Spinal Cord Cavernous Malformations,” Brain 146, no. 9 (2023): 3634–3647. 10.1093/brain/awad104 PMC10473567 36995941

[7] J. Weng , Y. Yang , D. Song , et al., “Somatic MAP3K3 Mutation Defines a Subclass of Cerebral Cavernous Malformation,” The American Journal of Human Genetics 108, no. 5 (2021): 942–950. 10.1016/j.ajhg.2021.04.005 PMC8206158 33891857

[8] T. Hong , X. Xiao , J. Ren , et al., “Somatic MAP3K3 and PIK3CA Mutations in Sporadic Cerebral and Spinal Cord Cavernous Malformations,” Brain 144, no. 9 (2021): 2648–2658. 10.1093/brain/awab117 33729480

[9] A. A. Ren , D. A. Snellings , Y. S. Su , et al., “PIK3CA and CCM Mutations Fuel Cavernomas Through a Cancer‐Like Mechanism,” Nature 594, no. 7862 (2021): 271–276. 10.1038/s41586-021-03562-8 PMC8626098 33910229

[10] M. Fu , Y. Hu , T. Lan , K. L. Guan , T. Luo , and M. Luo , “The Hippo Signalling Pathway and Its Implications in human Health and Diseases,” Signal Transduction and Targeted Therapy 7, no. 1 (2022): 376. 10.1038/s41392-022-01191-9 PMC9643504 36347846

[11] S. Ma , Z. Meng , R. Chen , and K. L. Guan , “The Hippo Pathway: Biology and Pathophysiology,” Annual Review of Biochemistry 88 (2019): 577–604. 10.1146/annurev-biochem-013118-111829 30566373

[12] R. Fan , N. G. Kim , and B. M. Gumbiner , “Regulation of Hippo Pathway by Mitogenic Growth Factors via Phosphoinositide 3‐kinase and Phosphoinositide‐dependent Kinase‐1,” Proceedings of the National Academy of Sciences of the United States of America 110, no. 7 (2013): 2569–2574. 10.1073/pnas.1216462110 PMC3574943 23359693

[13] T. Azad , H. J. Janse Van Rensburg , E. D. Lightbody , et al., “A LATS Biosensor Screen Identifies VEGFR as a Regulator of the Hippo Pathway in Angiogenesis,” Nature Communications 9, no. 1 (2018): 1061. 10.1038/s41467-018-03278-w PMC5849716 29535383

[14] F. X. Yu , B. Zhao , and K. L. Guan , “Hippo Pathway in Organ Size Control, Tissue Homeostasis, and Cancer,” Cell 163, no. 4 (2015): 811–828. 10.1016/j.cell.2015.10.044 PMC4638384 26544935

[15] A. Dey , X. Varelas , and K. L. Guan , “Targeting the Hippo Pathway in Cancer, Fibrosis, Wound Healing and Regenerative Medicine,” Nature Reviews Drug Discovery 19, no. 7 (2020): 480–494. 10.1038/s41573-020-0070-z PMC7880238 32555376

[16] J. Kim , Y. H. Kim , J. Kim , et al., “YAP/TAZ Regulates Sprouting Angiogenesis and Vascular Barrier Maturation,” Journal of Clinical Investigation 127, no. 9 (2017): 3441–3461. 10.1172/JCI93825 PMC5669570 28805663

[17] S. Kobayashi , A. G. Cox , K. F. Harvey , and B. M. Hogan , “Vasculature Is Getting Hip(po): Hippo Signaling in Vascular Development and Disease,” Developmental Cell 58, no. 23 (2023): 2627–2640. 10.1016/j.devcel.2023.11.002 38052179

[18] A. J. Haas , M. Karakus , C. Zihni , M. S. Balda , and K. Matter , “ZO‐1 Regulates Hippo‐Independent YAP Activity and Cell Proliferation via a GEF‐H1‐ and TBK1‐Regulated Signalling Network,” Cells 13, no. 7 (2024): 640. 10.3390/cells13070640 PMC11011562 38607079

[19] J. Lu , Z. Hu , Y. Deng , Q. Wu , M. Wu , and H. Song , “MEKK2 and MEKK3 Orchestrate Multiple Signals to Regulate Hippo Pathway,” Journal of Biological Chemistry 296 (2021): 100400. 10.1016/j.jbc.2021.100400 PMC7948509 33571521

[20] R. Cuttano , N. Rudini , L. Bravi , et al., “KLF4 is a Key Determinant in the Development and Progression of Cerebral Cavernous Malformations,” EMBO Molecular Medicine 8, no. 1 (2016): 6–24. 10.15252/emmm.201505433 PMC4718159 26612856

[21] G. Boulday , N. Rudini , L. Maddaluno , et al., “Developmental Timing of CCM2 Loss Influences Cerebral Cavernous Malformations in Mice,” Journal of Experimental Medicine 208, no. 9 (2011): 1835–1847. 10.1084/jem.20110571 PMC3171098 21859843

[22] A. T. Tang , K. R. Sullivan , C. C. Hong , et al., “Distinct Cellular Roles for PDCD10 Define a Gut‐Brain Axis in Cerebral Cavernous Malformation,” Science Translational Medicine 11, no. 520 (2019): 3521. 10.1126/scitranslmed.aaw3521 PMC6937779 31776290

[23] M. Peyre , D. Miyagishima , F. Bielle , et al., “Somatic PIK3CA Mutations in Sporadic Cerebral Cavernous Malformations,” New England Journal of Medicine 385, no. 11 (2021): 996–1004. 10.1056/NEJMoa2100440 PMC8606022 34496175

[24] D. Zhou , C. Conrad , F. Xia , et al., “Mst1 and Mst2 Maintain Hepatocyte Quiescence and Suppress Hepatocellular Carcinoma Development Through Inactivation of the Yap1 Oncogene,” Cancer Cell 16, no. 5 (2009): 425–438. 10.1016/j.ccr.2009.09.026 PMC3023165 19878874

[25] N. Kastan , K. Gnedeva , T. Alisch , et al., “Small‐Molecule Inhibition of Lats Kinases May Promote Yap‐Dependent Proliferation in Postmitotic Mammalian Tissues,” Nature Communications 12, no. 1 (2021): 3100. 10.1038/s41467-021-23395-3 PMC8149661 34035288

[26] L. Geng , T. Jiang , Y. Zhu , et al., “Identification of a Novel LATS1 Variant Associated With Familial Cerebral Cavernous Malformations in a Chinese family,” Neurological Sciences 43, no. 11 (2022): 6389–6397. 10.1007/s10072-022-06323-1 35986120

[27] M. Imajo , M. Ebisuya , and E. Nishida , “Dual Role of YAP and TAZ in Renewal of the Intestinal Epithelium,” Nature Cell Biology 17, no. 1 (2015): 7–19. 10.1038/ncb3084 25531778

[28] E. Borisova , K. Nishimura , Y. An , et al., “Structurally‐Discovered KLF4 Variants Accelerate and Stabilize Reprogramming to Pluripotency,” Iscience 25, no. 1 (2022): 103525. 10.1016/j.isci.2021.103525 PMC8786646 35106457

[29] H. Hashimoto , D. Wang , A. N. Steves , et al., “Distinctive Klf4 Mutants Determine Preference for DNA Methylation Status,” Nucleic Acids Res 44, no. 21 (2016): 10177–10185. 10.1093/nar/gkw774 PMC5137437 27596594

[30] Z. Li , B. Zhao , P. Wang , et al., “Structural Insights Into the YAP and TEAD Complex,” Genes & Development 24, no. 3 (2010): 235–240. 10.1101/gad.1865810 PMC2811825 20123905

[31] L. M. Chen , P. G. Loh , and H. W. Song , “Structural and Functional Insights Into the TEAD‐YAP Complex in the Hippo Signaling Pathway,” Protein & Cell 1, no. 12 (2010): 1073–1083. 10.1007/s13238-010-0138-3 PMC4728155 21213102

[32] M. O. Kim , S. H. Kim , Y. Y. Cho , et al., “ERK1 and ERK2 Regulate Embryonic Stem Cell Self‐renewal Through Phosphorylation of Klf4,” Nature Structural & Molecular Biology 19, no. 3 (2012): 283–290. 10.1038/nsmb.2217 22307056

[33] T. Moroishi , T. Hayashi , W. W. Pan , et al., “The Hippo Pathway Kinases LATS1/2 Suppress Cancer Immunity,” Cell 167, no. 6 (2016): 1525–1539.e17. 10.1016/j.cell.2016.11.005 PMC5512418 27912060

[34] Y. Zhao , T. Montminy , T. Azad , et al., “PI3K Positively Regulates YAP and TAZ in Mammary Tumorigenesis Through Multiple Signaling Pathways,” Molecular Cancer Research 16, no. 6 (2018): 1046–1058. 10.1158/1541-7786.MCR-17-0593 29545474

[35] C. C. Lai , B. Nelsen , E. Frias‐Anaya , et al., “Neuroinflammation Plays a Critical Role in Cerebral Cavernous Malformation Disease,” Circulation Research 131, no. 11 (2022): 909–925. 10.1161/CIRCRESAHA.122.321129 PMC9669201 36285625

[36] D. Wang , Y. Zhang , X. Xu , et al., “YAP Promotes the Activation of NLRP3 Inflammasome via Blocking K27‐Linked Polyubiquitination of NLRP3,” Nature Communications 12, no. 1 (2021): 2674. 10.1038/s41467-021-22987-3 PMC8113592 33976226

[37] Y. Lv , K. Kim , Y. Sheng , et al., “YAP Controls Endothelial Activation and Vascular Inflammation through TRAF6,” Circulation Research 123, no. 1 (2018): 43–56. 10.1161/CIRCRESAHA.118.313143 PMC6014930 29794022

[38] X. Cai , K. C. Wang , and Z. Meng , “Mechanoregulation of YAP and TAZ in Cellular Homeostasis and Disease Progression,” Frontiers in Cell and Developmental Biology 9 (2021): 673599. 10.3389/fcell.2021.673599 PMC8182050 34109179

[39] R. J. Dekker , S. Van Soest , R. D. Fontijn , et al., “Prolonged Fluid Shear Stress Induces a Distinct Set of Endothelial Cell Genes, Most Specifically Lung KrüPpel‐Like Factor (KLF2),” Blood 100, no. 5 (2002): 1689–1698. 10.1182/blood-2002-01-0046 12176889

[40] Q. Zheng , Y. Zou , P. Teng , et al., “Mechanosensitive Channel PIEZO1 Senses Shear Force to Induce KLF2/4 Expression via CaMKII/MEKK3/ERK5 Axis in Endothelial Cells,” Cells 11, no. 14 (2022): 2191. 10.3390/cells11142191 PMC9317998 35883633

[41] O. S. Fisher , H. Deng , D. Liu , et al., “Structure and Vascular Function of MEKK3–Cerebral Cavernous Malformations 2 Complex,” Nature Communications 6 (2015): 7937. 10.1038/ncomms8937 PMC4526114 26235885

[42] B. Lant , B. Yu , M. Goudreault , et al., “CCM‐3/STRIPAK Promotes Seamless Tube Extension Through Endocytic Recycling,” Nature Communications 6 (2015): 6449. 10.1038/ncomms7449 25743393

[43] G. Liu , Y. Qiu , N. You , et al., “Pre‐Ischaemic Empagliflozin Treatment Attenuates Blood–Brain Barrier Disruption via β‐Catenin Mediated Protection of Cerebral Endothelial Cells,” Cardiovascular Research 121, no. 5 (2025): 788–802. 10.1093/cvr/cvaf026 40173314

