# Supplementary material for: YAP Fuels Cerebral Cavernous Malformations by Regulating KLF4 Transcriptional Activity and Protein Stability
Source: MedComm (2020). 2026 Oct 23;7(11):e71086. doi: 10.1002/mco2.71086 (PMC13693918; doi:10.1002/mco2.71086)
Supplement: Supplementary file 1 [file MCO2-7-e71086-s001.pdf]

YAP flames cerebral cavernous malformations by regulating KLF4 transcriptional activity and protein stability

Tingting Ye[1†], Qi Zheng[4†], Zonghao Hu[1†], Haonan Fan[1], Zhongxing Sun[1], Xiya Li[1], Haomin Mo[1], Chengdong Chang[5], Yanhua Xu[1], Kai Wang[2,3*], Dong Fang[1*], Hai Song[2,3,6,7*]

1. Life Sciences Institute, Zhejiang University, Hangzhou, Zhejiang, 310058, China

2. Department of Respiratory and Critical Care Medicine, Center for Oncology Medicine, the Fourth Affiliated Hospital of School of Medicine, and International School of Medicine, International Institutes of Medicine, Zhejiang University, Yiwu, Zhejiang, 322000, China

3. Zhejiang Key Laboratory of Precision Diagnosis and Treatment for Lung Cancer, Yiwu, Zhejiang, 322000, China

4. Department of Cardiovascular Surgery, the First Affiliated Hospital, School of Medicine, Zhejiang University, Hangzhou, Zhejiang, 310009, China

5. Department of Pathology, The First Affiliated Hospital, School of Medicine, Zhejiang University, Hangzhou, Zhejiang, 310009, China

6. The MOE Key Laboratory of Biosystems Homeostasis & Protection, Zhejiang University, Hangzhou, Zhejiang, 310058, China

7. State Key Laboratory of Transvascular Implantation Devices, Hangzhou, 310009, China.

*Correspondence: haisong@zju.edu.cn (H.S.), dfang@zju.edu.cn (D.F.), kaiw@zju.edu.cn (K.W.)

[†]These authors contributed equally to this work.

Supplementary Materials include:

Supplementary Figures (Figs. S1–S11)

Supplementary Methods

Supplementary Tables (S1-S3)

Fig. S1

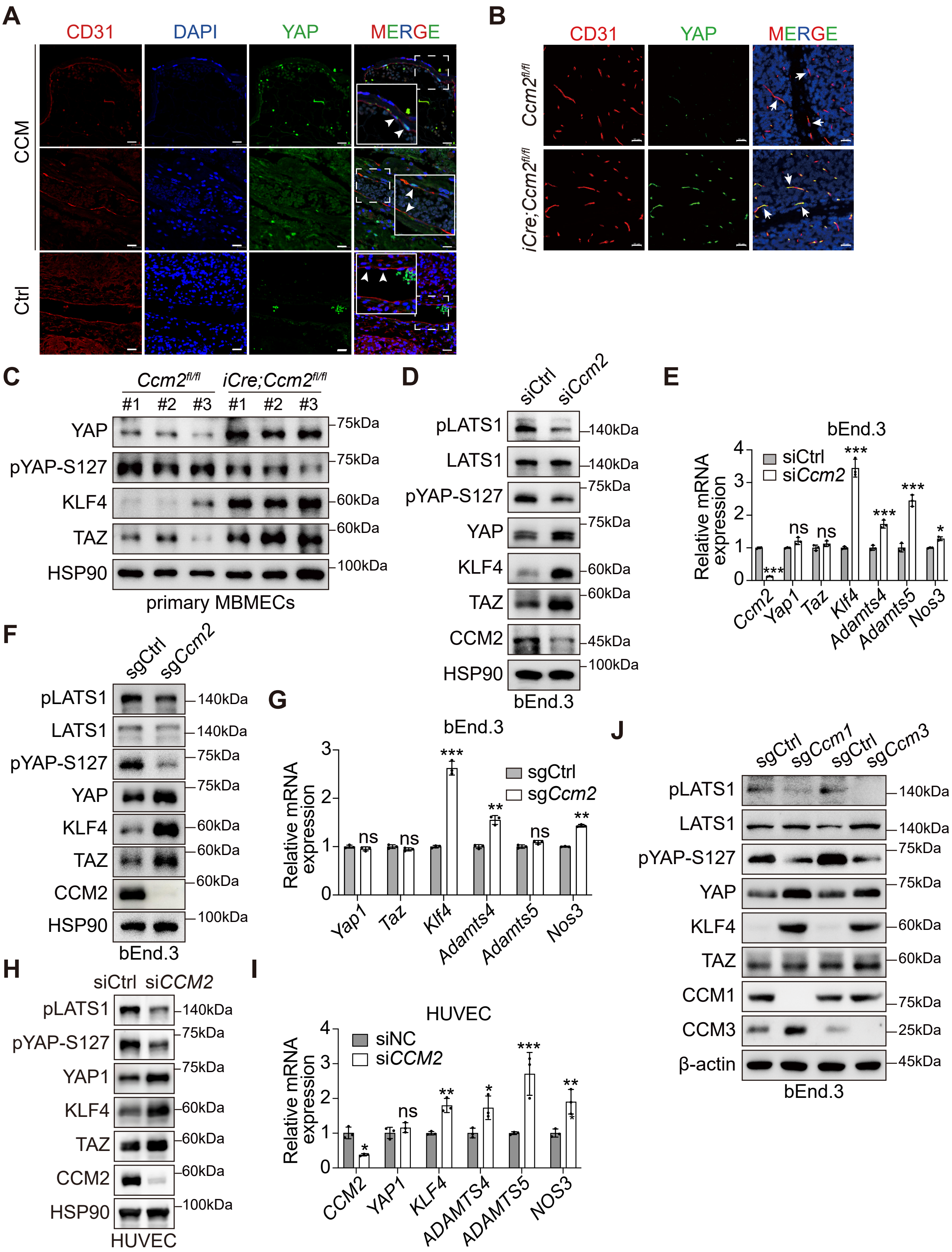
A
CD31
DAPI
YAP
MERGE
CCM
Ctrl
B
CD31
YAP
MERGE
Ccm2fl/fl
iCre;Ccm2fl/fl
C
Ccm2fl/fl
iCre;Ccm2fl/fl
#1 #2 #3 #1 #2 #3
YAP
pYAP-S127
KLF4
TAZ
HSP90
75kDa
75kDa
60kDa
60kDa
100kDa
primary MBMECs
D
siCtrl
siCcm2
pLATS1
LATS1
pYAP-S127
YAP
KLF4
TAZ
CCM2
HSP90
140kDa
140kDa
75kDa
75kDa
60kDa
60kDa
45kDa
100kDa
bEnd.3
E
bEnd.3
Relative mRNA expression
siCtrl
siCcm2
***
ns
ns
***
***
***
*
0 1 2 3 4
Ccm2 Yap1 Taz Klf4 Adamts4 Adamts5 Nos3
F
sgCtrl
sgCcm2
pLATS1
LATS1
pYAP-S127
YAP
KLF4
TAZ
CCM2
HSP90
140kDa
140kDa
75kDa
75kDa
60kDa
60kDa
60kDa
100kDa
bEnd.3
G
bEnd.3
Relative mRNA expression
sgCtrl
sgCcm2
ns
ns
***
**
ns
**
0 1 2 3
Yap1 Taz Klf4 Adamts4 Adamts5 Nos3
H
siCtrl
siCCM2
pLATS1
pYAP-S127
YAP1
KLF4
TAZ
CCM2
HSP90
140kDa
75kDa
75kDa
60kDa
60kDa
60kDa
100kDa
HUVEC
I
HUVEC
Relative mRNA expression
siNC
siCCM2
*
ns
**
*
***
**
0 1 2 3 4
CCM2 YAP1 KLF4 ADAMTS4 ADAMTS5 NOS3
J
sgCtrl
sgCcm1
sgCtrl
sgCcm3
pLATS1
LATS1
pYAP-S127
YAP
KLF4
TAZ
CCM1
CCM3
β-actin
140kDa
140kDa
75kDa
75kDa
60kDa
60kDa
75kDa
25kDa
45kDa
bEnd.3

Figure S1. CCM gene deletion increases YAP protein levels.

A. Representative immunofluorescence images showing YAP (green) and the vascular endothelial marker CD31 (red) in surgically resected tissues from 30 CCM patients and 20 control tissues. Nuclei were counterstained with DAPI (blue). Arrows indicate YAP signals in CD31-positive vascular ECs. Scale bars: 20 μm. **B.** Representative immunofluorescence staining of YAP (green) and CD31 (red) in the cerebral vascular endothelium of adult *Ccm2*$^{fl/fl}$ and *iCre*;*Ccm2*$^{fl/fl}$ mice. n=3 mice per genotype. Scale bars: 20 μm. **C.** Western blot analysis of YAP and KLF4 protein levels in *Ccm2*-deficient primary MBMECs. **D.** Western blot analysis of YAP and KLF4 protein levels in *Ccm2*-knockdown bEnd.3 cells. **E.** RT-qPCR analysis of *Klf4* mRNA levels and its downstream target genes in *Ccm2*-knockdown bEnd.3 cells (n = 3 independent experiments). **F.** Western blot analysis of YAP and KLF4 protein levels in *Ccm2*-knockout bEnd.3 cells. **G.** RT-qPCR analysis of *Klf4* mRNA levels and its downstream target genes in *Ccm2*-knockout bEnd.3 cells (n = 3 independent experiments). **H.** Western blot analysis of YAP and KLF4 protein levels in *Ccm2*-knockdown HUVEC. **I.** RT-qPCR analysis of *Klf4* mRNA levels and its downstream target genes in *Ccm2*-knockdown HUVEC (n = 3 independent experiments). **J.** Western blot analysis of YAP and KLF4 protein levels in *Ccm1*- or *Ccm3*-knockout bEnd.3 cells. Statistical analyses were performed using two-way ANOVA followed by Tukey's multiple comparisons test (E, G, I). Data are shown as means ± SEM; n.s., not significant, $P > 0.05$; $^*P < 0.05$; $^{**}P < 0.01$; $^{***}P < 0.001$.

Fig. S2

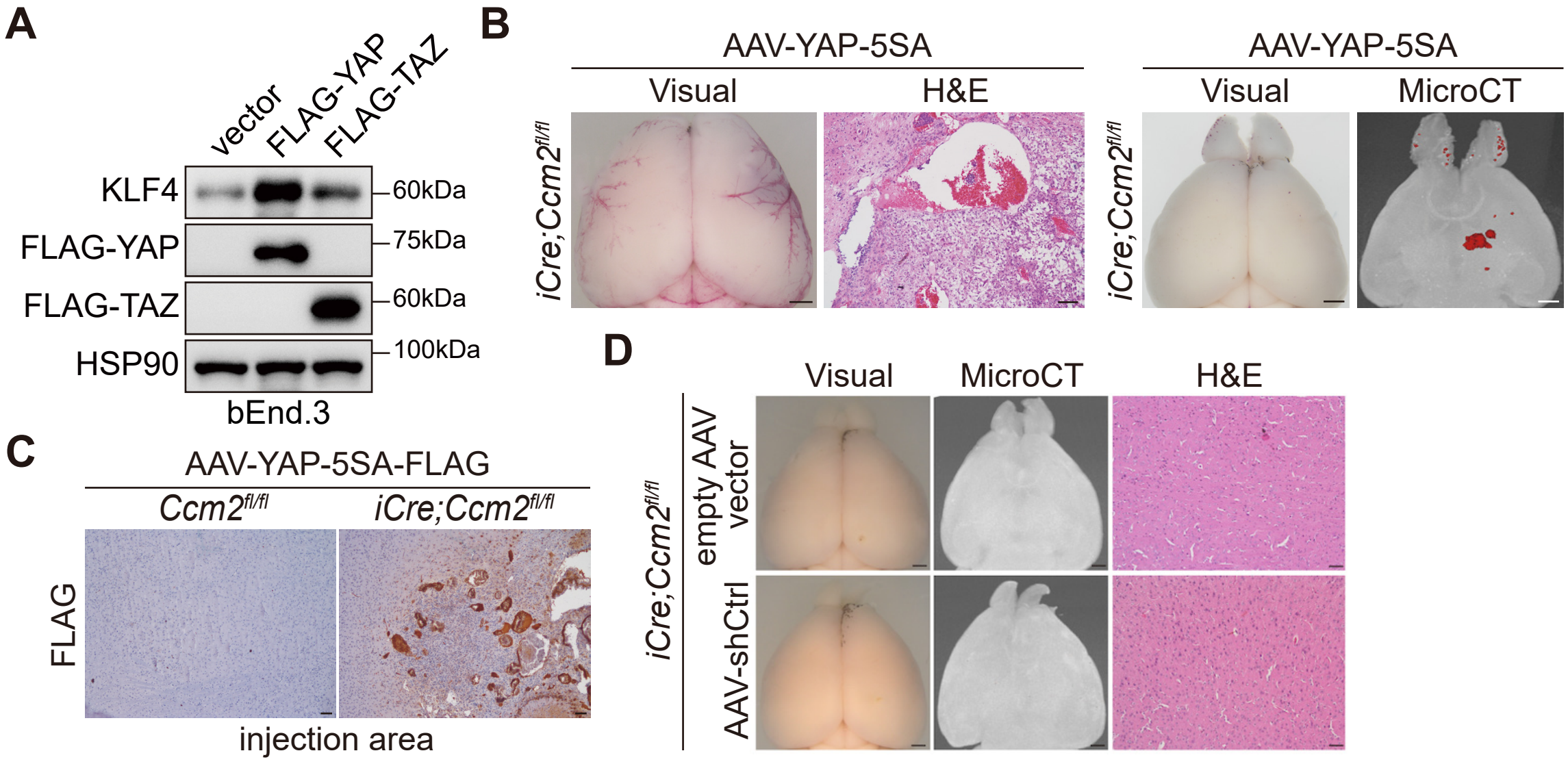

A
vector
FLAG-YAP
FLAG-TAZ
KLF4
FLAG-YAP
FLAG-TAZ
HSP90
60kDa
75kDa
60kDa
100kDa
bEnd.3
B
AAV-YAP-5SA
Visual
H&E
iCre;Ccm2fl/fl
AAV-YAP-5SA
Visual
MicroCT
iCre;Ccm2fl/fl
C
AAV-YAP-5SA-FLAG
Ccm2fl/fl
iCre;Ccm2fl/fl
FLAG
injection area
D
Visual
MicroCT
H&E
iCre;Ccm2fl/fl
empty AAV vector
AAV-shCtrl

Figure S2. YAP activation promotes CCM progression.

A. Western blot analysis of KLF4 protein levels in bEnd.3 cells overexpressing YAP or TAZ via lentiviral transduction. **B.** Representative visual, micro-CT and H&E images of brains collected 30 days following intracranial injection of YAP-5SA-expressing AAV into adult *iCre*;*Ccm2*$^{fl/fl}$ mice. Scale bars: 1 mm (visual and micro-CT images); 50 μm (H&E images). **C.** DAB staining for the FLAG tag confirmed successful infection of the mouse brain by AAV-YAP-5SA-FLAG.

Fig. S3

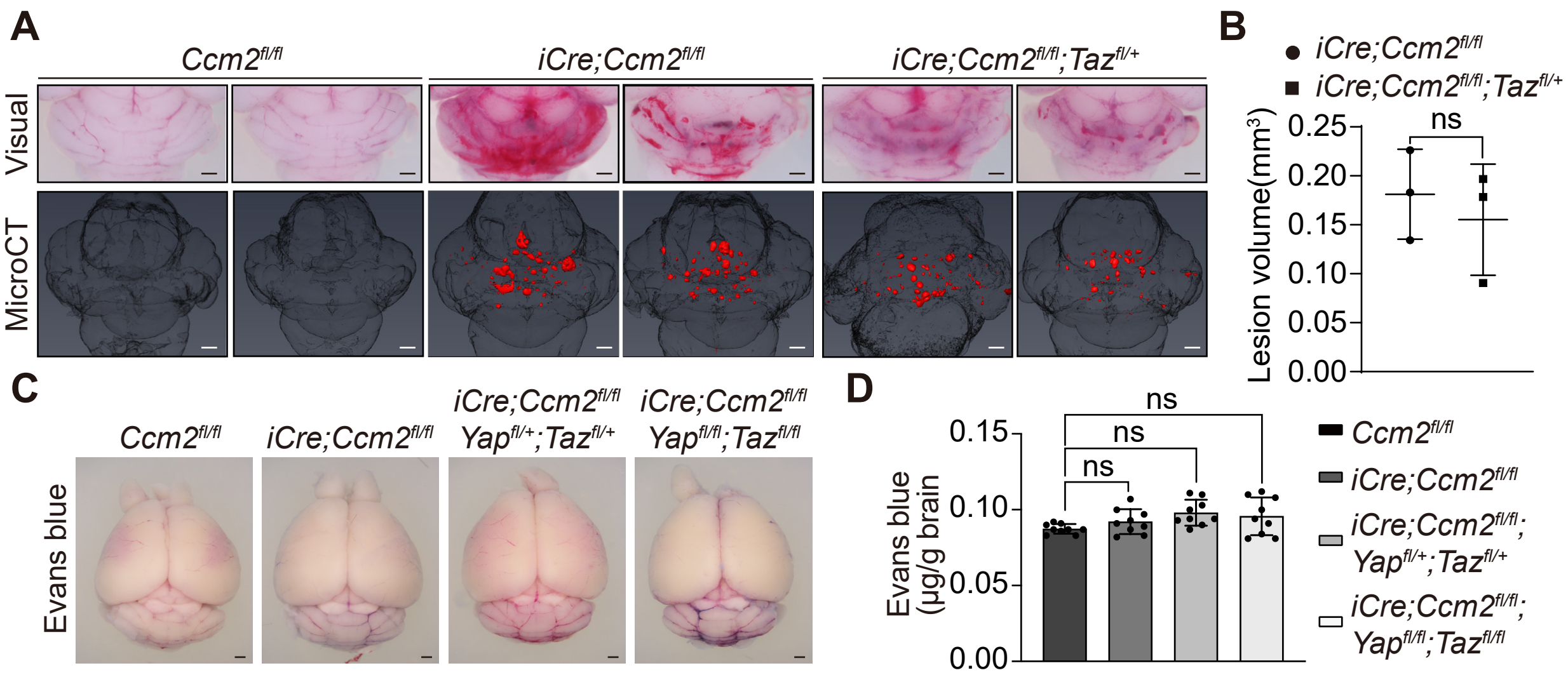

A
Ccm2fl/fl
iCre;Ccm2fl/fl
iCre;Ccm2fl/fl;Tazfl/+
Visual
MicroCT
B
iCre;Ccm2fl/fl
iCre;Ccm2fl/fl;Tazfl/+
ns
Lesion volume(mm3)
0.25
0.20
0.15
0.10
0.05
0.00
C
Ccm2fl/fl
iCre;Ccm2fl/fl
iCre;Ccm2fl/fl Yapfl/+;Tazfl/+
iCre;Ccm2fl/fl Yapfl/fl;Tazfl/fl
Evans blue
D
ns
ns
ns
Evans blue (μg/g brain
0.15
0.10
0.05
0.00
Ccm2fl/fl
iCre;Ccm2fl/fl
iCre;Ccm2fl/fl; Yapfl/+;Tazfl/+
iCre;Ccm2fl/fl; Yapfl/fl;Tazfl/fl

Figure S3. Endothelial deletion of *Taz* has little effect on CCM progression.

A. Representative visual (top) and micro-CT (bottom) images of the P14 brains from *Ccm2*$^{fl/fl}$, *iCre*;*Ccm2*$^{fl/fl}$, *iCre*;*Ccm2*$^{fl/fl}$;*Taz*$^{fl/+}$, and *iCre*;*Ccm2*$^{fl/fl}$;*Taz*$^{fl/fl}$ mice. Scale bars: 1 mm. **B.** Quantification of lesion volumes by micro-CT analysis at P14 (n = 3 mice per genotype). **C.** Representative images of Evans blue extravasation in fresh whole-brain tissues from adult *Ccm2*$^{fl/fl}$, *iCre*;*Ccm2*$^{fl/fl}$;*Yap*$^{fl/+}$;*Taz*$^{fl/+}$ and *iCre*;*Ccm2*$^{fl/fl}$;*Yap*$^{fl/fl}$;*Taz*$^{fl/fl}$ mice. Scale bars: 1 mm. **D.** Quantification of Evans blue extravasation in brain of the indicated adult mice (n=9 mice per genotype). Statistical analyses were performed using one-way ANOVA followed by Dunnett's multiple comparison test (B, D). Data are shown as means ± SEM; n.s., not significant, $P > 0.05$.

Fig. S4

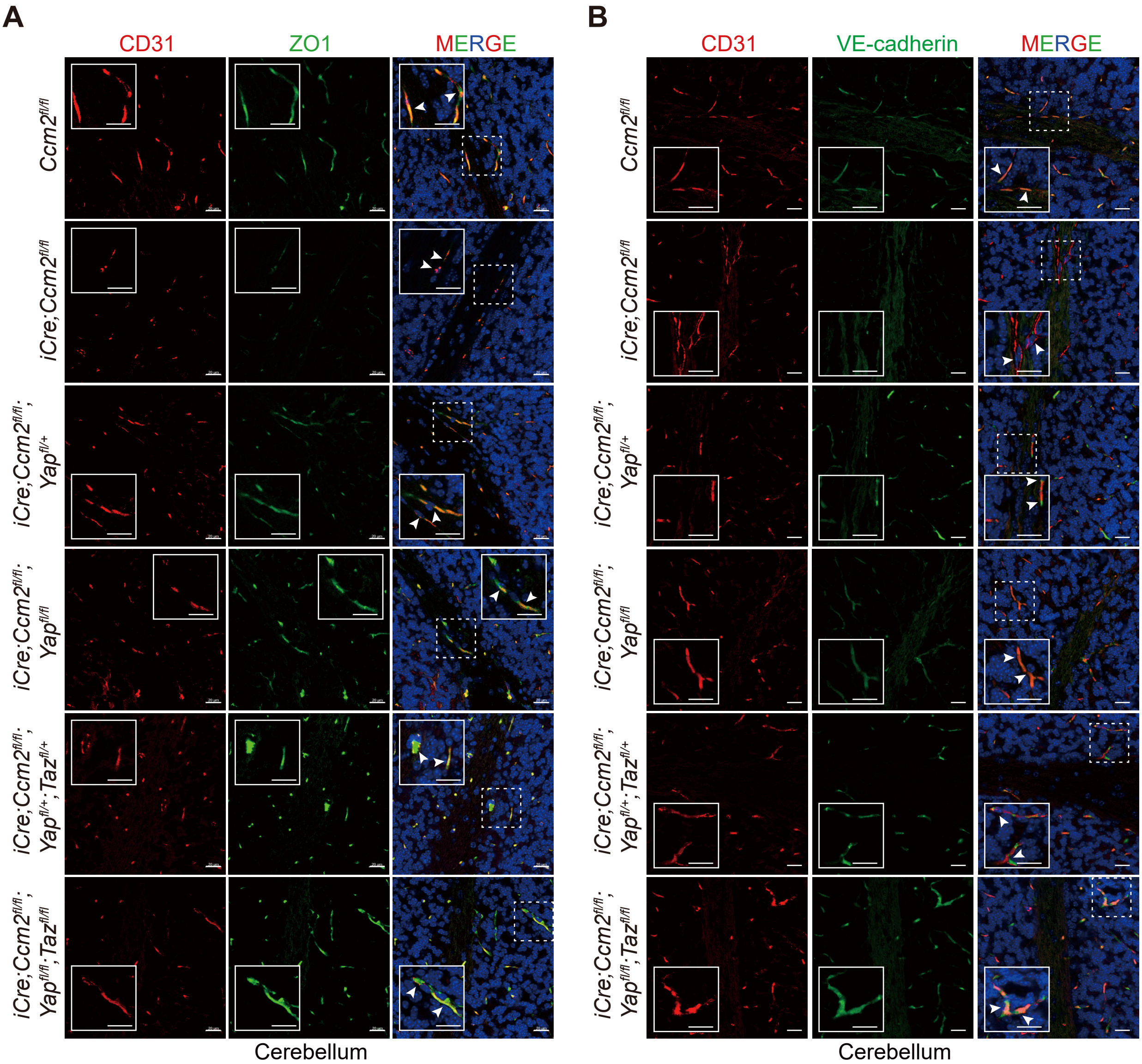
A
CD31
ZO1
MERGE
Ccm2fl/fl
iCre;Ccm2fl/fl
iCre;Ccm2fl/fl;
Yapfl/+
iCre;Ccm2fl/fl;
Yapfl/fl
iCre;Ccm2fl/fl;
Yapfl/+;Tazfl/+
iCre;Ccm2fl/fl;
Yapfl/fl;Tazfl/fl
Cerebellum
B
CD31
VE-cadherin
MERGE
Ccm2fl/fl
iCre;Ccm2fl/fl
iCre;Ccm2fl/fl;
Yapfl/+
iCre;Ccm2fl/fl;
Yapfl/fl
iCre;Ccm2fl/fl;
Yapfl/+;Tazfl/+
iCre;Ccm2fl/fl;
Yapfl/fl;Tazfl/fl
Cerebellum

Figure S4. Endothelial deletion of *Yap* repairs TJs and AJs in the cerebellar microvessels disrupted by *Ccm2* dificient.

A. Immunofluorescence staining of TJs (marked by ZO-1) and CD31 in cerebellar microvessels from adult *Ccm2*$^{fl/fl}$, *iCre*;*Ccm2*$^{fl/fl}$, *iCre*;*Ccm2*$^{fl/fl}$;*Yap*$^{fl/+}$ and *iCre*;*Ccm2*$^{fl/fl}$;*Yap*$^{fl/+}$;*Taz*$^{fl/+}$ mice. White arrows indicate ZO-1 signals in vascular ECs. n=3 mice per genotype. Scale bars: 20 μm. **B.** Immunofluorescence staining of AJs (marked by VE-cadherin) and CD31 in cerebellar microvessels of the indicated adult mice. White arrows indicate VE-cadherin signals in vascular ECs. n=3 mice per genotype. Scale bars: 20 μm.

Fig. S5

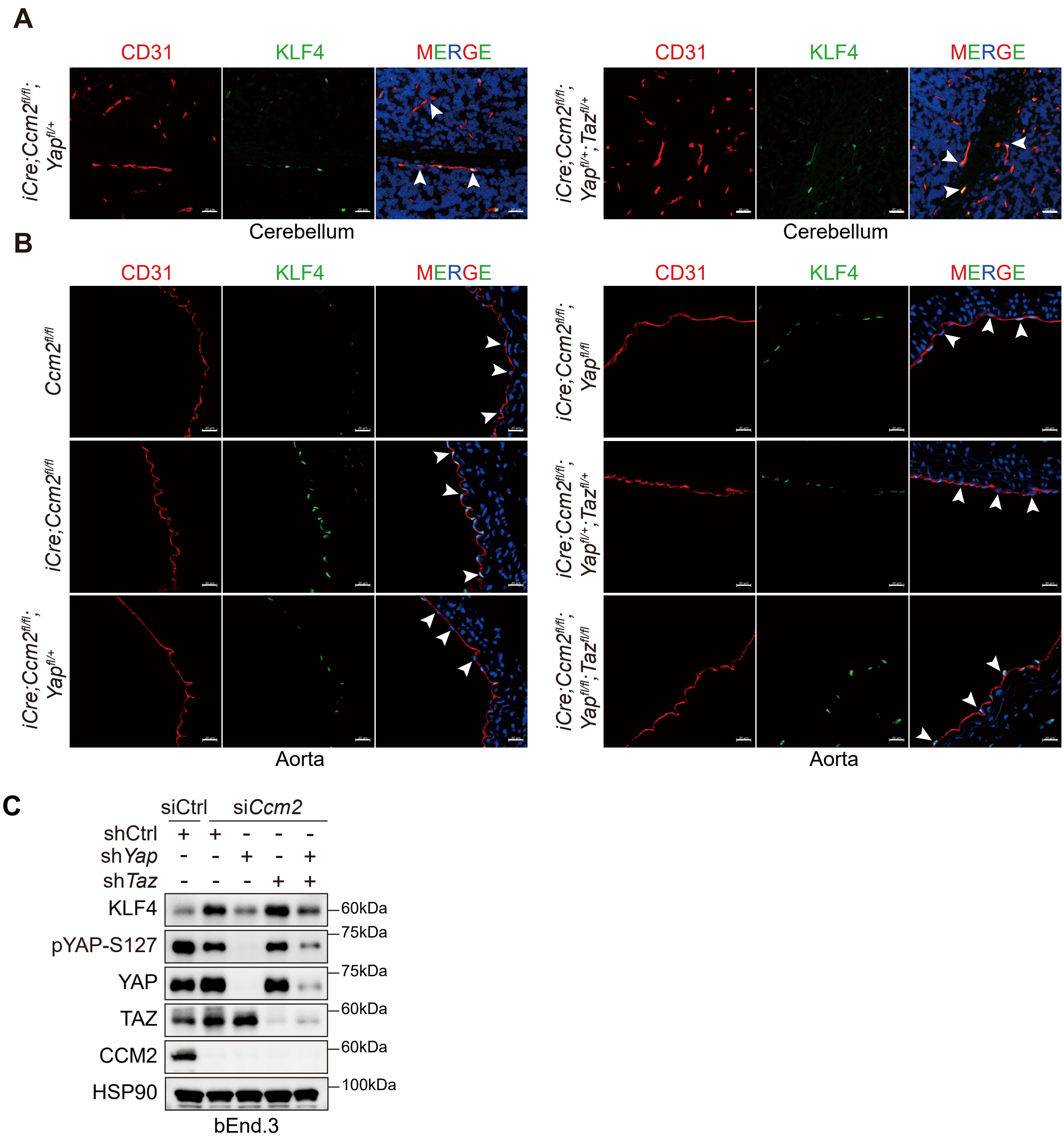
A
CD31
KLF4
MERGE
iCre;Ccm2fl/fl;
Yapfl/+
iCre;Ccm2fl/fl;
Yapfl/+;Tazfl/+
Cerebellum
Cerebellum
B
CD31
KLF4
MERGE
Ccm2fl/fl
iCre;Ccm2fl/fl
iCre;Ccm2fl/fl;
Yapfl/+
iCre;Ccm2fl/fl;
Yapfl/fl
iCre;Ccm2fl/fl;
Yapfl/+;Tazfl/+
iCre;Ccm2fl/fl;
Yapfl/fl;Tazfl/fl
Aorta
Aorta
C
siCtrl
siCcm2
shCtrl
shYap
shTaz
KLF4
pYAP-S127
YAP
TAZ
CCM2
HSP90
60kDa
75kDa
75kDa
60kDa
60kDa
100kDa
bEnd.3

Figure S5. *Yap* deletion reduces endothelial KLF4 expression induced by *Ccm2* dificiency.

A. Immunofluorescence staining of KLF4 (green) and CD31 (red) in the adult mouse cerebellum of *iCre*;*Ccm2*$^{fl/fl}$;*Yap*$^{fl/+}$ and *iCre*;*Ccm2*$^{fl/fl}$;*Yap*$^{fl/+}$;*Taz*$^{fl/+}$ mice. Nuclei were counterstained with DAPI (blue). White arrows indicate KLF4 signals in the cerebellar vascular endothelium. n=3 mice per genotype. Scale bars: 20 μm. **B.** Immunofluorescence staining of KLF4 and CD31 in adult aortas from the indicated adult mice. White arrows indicate KLF4 signals in aortic ECs. Scale bars: 20 μm. **C.** Western blot analysis of KLF4 protein levels in WT, *Yap*-knockdown, *Taz*-knockdown and *Yap/Taz*-double-knockdown bEnd.3 cells treated with si*Ccm2* or control siRNA.

Fig. S6

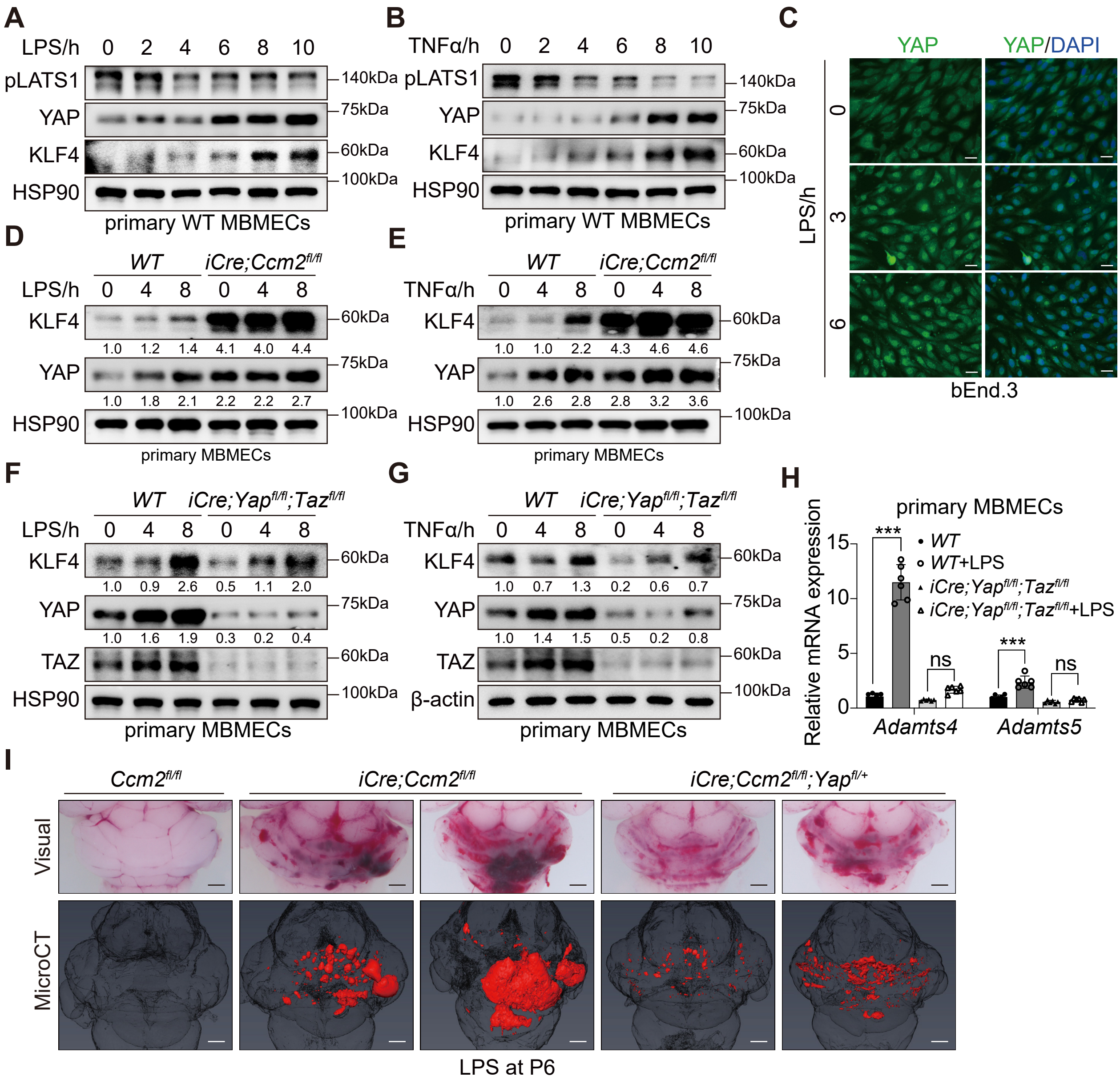

A
LPS/h 0 2 4 6 8 10
pLATS1
YAP
KLF4
HSP90
140kDa
75kDa
60kDa
100kDa
primary WT MBMECs
B
TNFα/h 0 2 4 6 8 10
pLATS1
YAP
KLF4
HSP90
primary WT MBMECs
C
YAP
YAP/DAPI
LPS/h
0
3
6
bEnd.3
D
WT
iCre;Ccm2fl/fl
LPS/h 0 4 8 0 4 8
KLF4
1.0 1.2 1.4 4.1 4.0 4.4
YAP
1.0 1.8 2.1 2.2 2.2 2.7
HSP90
primary MBMECs
E
WT
iCre;Ccm2fl/fl
TNFα/h 0 4 8 0 4 8
KLF4
1.0 1.0 2.2 4.3 4.6 4.6
YAP
1.0 2.6 2.8 2.8 3.2 3.6
HSP90
primary MBMECs
F
WT
iCre;Yapfl/fl;Tazfl/fl
LPS/h 0 4 8 0 4 8
KLF4
1.0 0.9 2.6 0.5 1.1 2.0
YAP
1.0 1.6 1.9 0.3 0.2 0.4
TAZ
HSP90
primary MBMECs
G
WT
iCre;Yapfl/fl;Tazfl/fl
TNFα/h 0 4 8 0 4 8
KLF4
1.0 0.7 1.3 0.2 0.6 0.7
YAP
1.0 1.4 1.5 0.5 0.2 0.8
TAZ
β-actin
primary MBMECs
H
primary MBMECs
Relative mRNA expression
15
10
5
0
WT
WT+LPS
iCre;Yapfl/fl;Tazfl/fl
iCre;Yapfl/fl;Tazfl/fl+LPS
***
ns
Adamts4
Adamts5
I
Ccm2fl/fl
iCre;Ccm2fl/fl
iCre;Ccm2fl/fl;Yapfl/+
Visual
MicroCT
LPS at P6

Figure S6. Endothelial deletion of *Yap* alleviates CCM formation under inflammatory stimulation.

A and B. Western blot analysis of YAP and KLF4 protein levels in WT primary MBMECs treated with LPS (A) or TNFα (B). Cells were treated with LPS (1 μg/mL) or TNFα (500 ng/mL) for 2, 4, 6, 8, or 10 h. **C.** Immunofluorescence staining of YAP (green) in LPS-treated bEnd.3 cells. Cells were treated with LPS (1 μg/mL) for 3 or 6 h. Nuclei were counterstained with DAPI (blue). Scale bars: 20 μm. **D and E.** Western blot analysis of YAP and KLF4 protein levels in primary MBMECs harvested from adult $Ccm2^{fl/fl}$ and $iCre;Ccm2^{fl/fl}$ mice treated with LPS (D) and TNFα (E). **F and G.** Western blot analysis of KLF4 protein levels in primary MBMECs harvested from adult $Yap^{fl/fl};Taz^{fl/fl}$ and $iCre;Yap^{fl/fl};Taz^{fl/fl}$ mice treated with LPS (F) and TNFα (G). LPS, 1 μg/mL; TNFα, 500 ng/mL. **H.** RT-qPCR analysis of *Adamts4* and *Adamts5* mRNA levels in LPS-treated primary MBMECs of the indicated genotypes. n=6 mice per genotype. **I.** Representative visual (top) and micro-CT (bottom) images of the indicated P12 brains. Neonatal mice were injected intraperitoneally with 20 μg tamoxifen on postnatal days 1 (P1) and 3 (P3). Mice were then injected intraperitoneally with LPS (5 μg) at P6 and euthanized at P12 for brain harvest. n = 5 mice per genotype. Scale bars: 1 mm. Statistical analyses were performed using two-way ANOVA followed by Tukey's multiple comparisons test (H). Data are shown as means ± SEM; n.s., not significant, $P > 0.05$; $^{***}P < 0.001$.

Fig S7

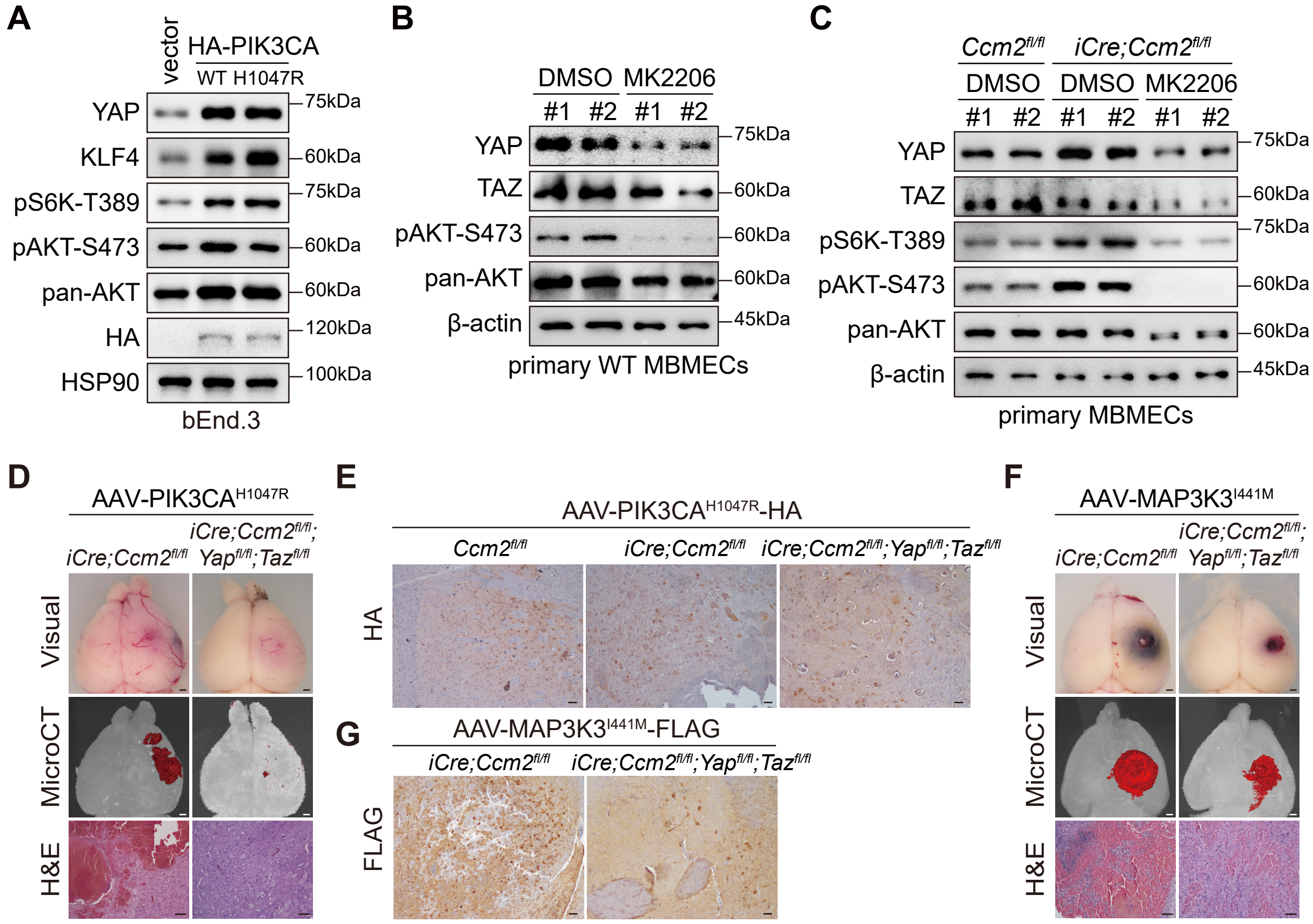
A
vector
HA-PIK3CA
WT H1047R
YAP
KLF4
pS6K-T389
pAKT-S473
pan-AKT
HA
HSP90
75kDa
60kDa
75kDa
60kDa
60kDa
120kDa
100kDa
bEnd.3
B
DMSO MK2206
#1 #2 #1 #2
YAP
TAZ
pAKT-S473
pan-AKT
β-actin
75kDa
60kDa
60kDa
60kDa
45kDa
primary WT MBMECs
C
Ccm2fl/fl iCre;Ccm2fl/fl
DMSO DMSO MK2206
#1 #2 #1 #2 #1 #2
YAP
TAZ
pS6K-T389
pAKT-S473
pan-AKT
β-actin
75kDa
60kDa
75kDa
60kDa
60kDa
45kDa
primary MBMECs
D
AAV-PIK3CAH1047R
iCre;Ccm2fl/fl
iCre;Ccm2fl/fl; Yapfl/fl;Tazfl/fl
Visual
MicroCT
H&E
E
AAV-PIK3CAH1047R-HA
Ccm2fl/fl
iCre;Ccm2fl/fl
iCre;Ccm2fl/fl;Yapfl/fl;Tazfl/fl
HA
G
AAV-MAP3K3I441M-FLAG
iCre;Ccm2fl/fl
iCre;Ccm2fl/fl;Yapfl/fl;Tazfl/fl
FLAG
F
AAV-MAP3K3I441M
iCre;Ccm2fl/fl
iCre;Ccm2fl/fl; Yapfl/fl;Tazfl/fl
Visual
MicroCT
H&E

Figure S7. Endothelial loss of *Yap/Taz* alleviates PIK3CA^{H1047R} and MAP3K3^{I441M} fueled CCMs.

A. Western blot analysis of YAP and KLF4 protein levels in bEnd.3 cells overexpressing PIK3CA or PIK3CA^{H1047R}. **B.** Western blot analysis of YAP and TAZ protein levels in primary MBMECs treated with MK-2206 (10 μM) for 24 h. **C.** Western blot analysis of YAP protein levels in *Ccm2*-knockout primary MBMECs treated with MK-2206 (10 μM) for 24 h. **D.** Representative visual (top), micro-CT (medium), and H&E (bottom) images of brains from adult *Ccm2*$^{fl/fl}$, *iCre*;*Ccm2*$^{fl/fl}$ and *iCre*;*Ccm2*$^{fl/fl}$;*Yap*$^{fl/fl}$;*Taz*$^{fl/fl}$ mice collected 30 days after intracranial injection of PIK3CA^{H1047R} AAV-BR1. Scale bars: 1 mm (visual and micro-CT images); 50 μm (H&E images). **E.** DAB staining for HA tags confirmed successful brain transduction by AAV-PIK3CA^{H1047R}-HA. Scale bars: 50 μm. **F.** Representative visual (top), micro-CT (medium) and H&E (bottom) images of brains from adult *iCre*;*Ccm2*$^{fl/fl}$ and *iCre*;*Ccm2*$^{fl/fl}$;*Yap*$^{fl/fl}$;*Taz*$^{fl/fl}$ mice collected 10 days after intracranial injection of MAP3K3^{I441M} AAV-BR1. Scale bars: 1 mm (visual and micro-CT images); 50 μm (H&E images). **G.** DAB staining for FLAG tags confirmed successful brain transduction by AAV-MAP3K3^{I441M}-FLAG. Scale bars: 50 μm.

Fig S8

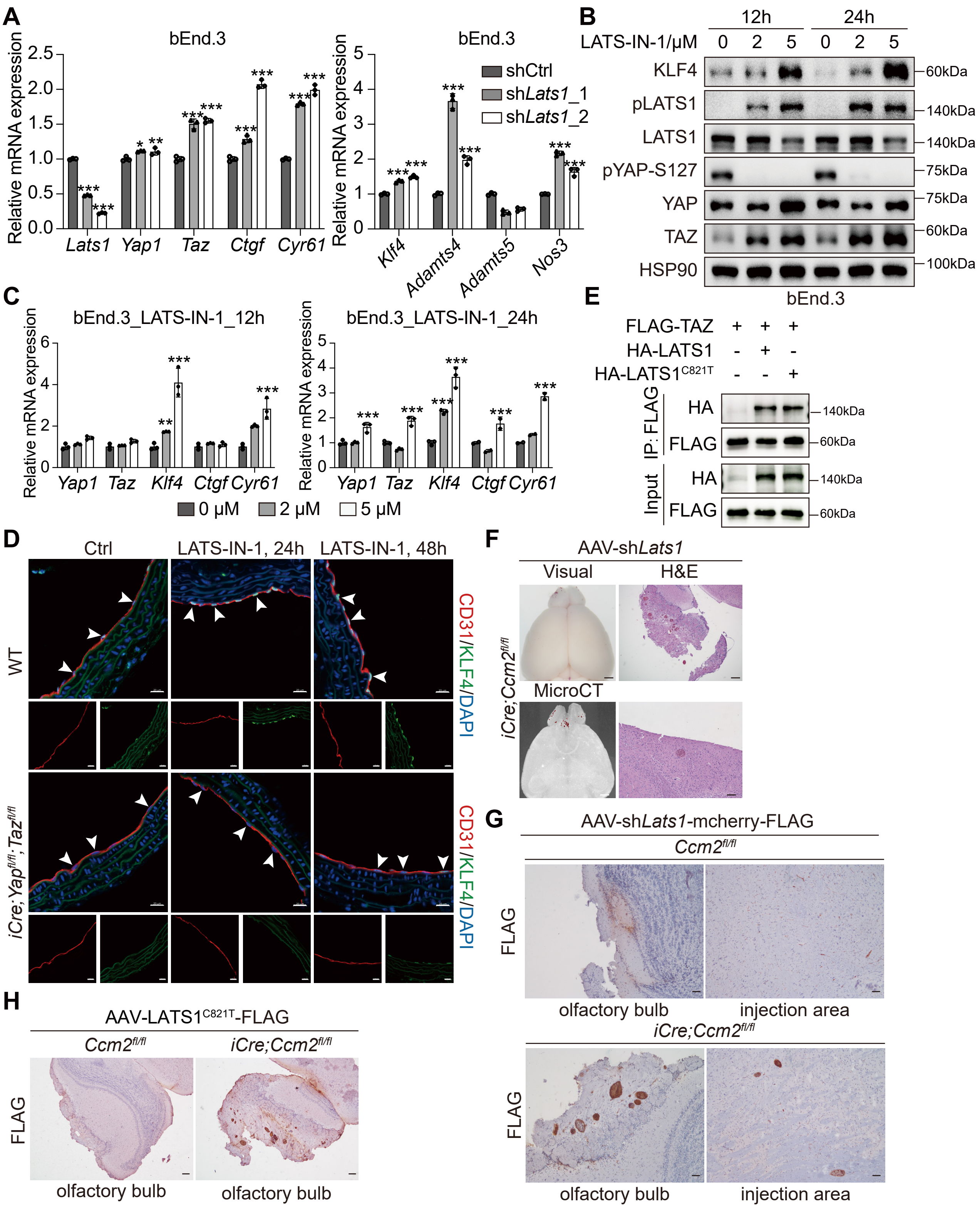
A
bEnd.3
Relative mRNA expression
Lats1
Yap1
Taz
Ctgf
Cyr61
Klf4
Adamts4
Adamts5
Nos3
shCtrl
shLats1_1
shLats1_2
B
12h
24h
LATS-IN-1/μM
KLF4
pLATS1
LATS1
pYAP-S127
YAP
TAZ
HSP90
60kDa
140kDa
75kDa
100kDa
bEnd.3
C
bEnd.3_LATS-IN-1_12h
bEnd.3_LATS-IN-1_24h
0 μM
2 μM
5 μM
E
FLAG-TAZ
HA-LATS1
HA-LATS1C821T
IP: FLAG
Input
HA
FLAG
D
Ctrl
LATS-IN-1, 24h
LATS-IN-1, 48h
WT
iCre;Yapfl/fl;Tazfl/fl
CD31/KLF4/DAPI
F
AAV-shLats1
Visual
H&E
MicroCT
iCre;Ccm2fl/fl
G
AAV-shLats1-mcherry-FLAG
Ccm2fl/fl
iCre;Ccm2fl/fl
FLAG
olfactory bulb
injection area
H
AAV-LATS1C821T-FLAG
Ccm2fl/fl
iCre;Ccm2fl/fl
FLAG
olfactory bulb

Figure S8. LATS inhibition increases endothelial KLF4 expression and promotes CCM progression.

A. RT-qPCR analysis of *Klf4* mRNA levels and its downstream target genes in *Lats1*-knockdown bEnd.3 cells (n=3 per group). **B.** Western blot analysis of YAP and KLF4 protein levels in bEnd.3 cells treated with LATS-IN-1. **C.** RT-qPCR analysis of *Cyr61* and *Ctgf* mRNA levels in bEnd.3 cells treated with LATS-IN-1 (n=3 per group). Cells were treated with LATS-IN-1 (2 μM and 5 μM) or DMSO for 12 h or 24 h, respectively. **D.** Immunofluorescence staining of KLF4 (green) and CD31 (red) in adult aorta following tail vein injection of LATS-IN-1. WT and *iCre*;$Yap^{fl/fl}$;$Taz^{fl/fl}$ mice received were three intraperitoneal injections of tamoxifen (100 μg) or corn oil every other day. Mice were then intravenously injected with either vehicle or 1 mg LATS-IN-1 in working solution twice within 48 hours. n = 5 mice per group. Scale bars: 20 μm. **E.** Co-IP analysis of the interaction between TAZ and LATS1 or $LATS1^{C821T}$. HEK293T cells were transiently co-transfected with FLAG-TAZ and HA- LATS1 or HA-$LATS1^{C821T}$ plasmids. FLAG-TAZ proteins were immunoprecipitated, and HA-LATS1 or HA-$LATS1^{C821T}$ proteins were detected by immunoblotting. **F.** Representative visual, micro-CT and H&E images of brains collected 60 days after injection of sh*Lats1*-expressing AAV into adult *iCre*;$Ccm2^{fl/fl}$ mice. Scale bars: 1 mm (visual and micro-CT images); 50 μm (H&E images). **G.** DAB staining for the FLAG tag confirmed successful infection of the mouse brain by AAV-sh*Lats1*-mcherry-FLAG. Scale bars: 50 μm. **H.** DAB staining for the FLAG tag confirmed successful infection of the mouse brain by AAV-$Lats1^{C821T}$-FLAG. Scale bars: 50 μm. Statistical analyses were performed using two-way ANOVA followed by Tukey's multiple comparisons test (A, C). Data are shown as means ± SEM; **$P < 0.01$; ***$P < 0.001$.

Fig. S9

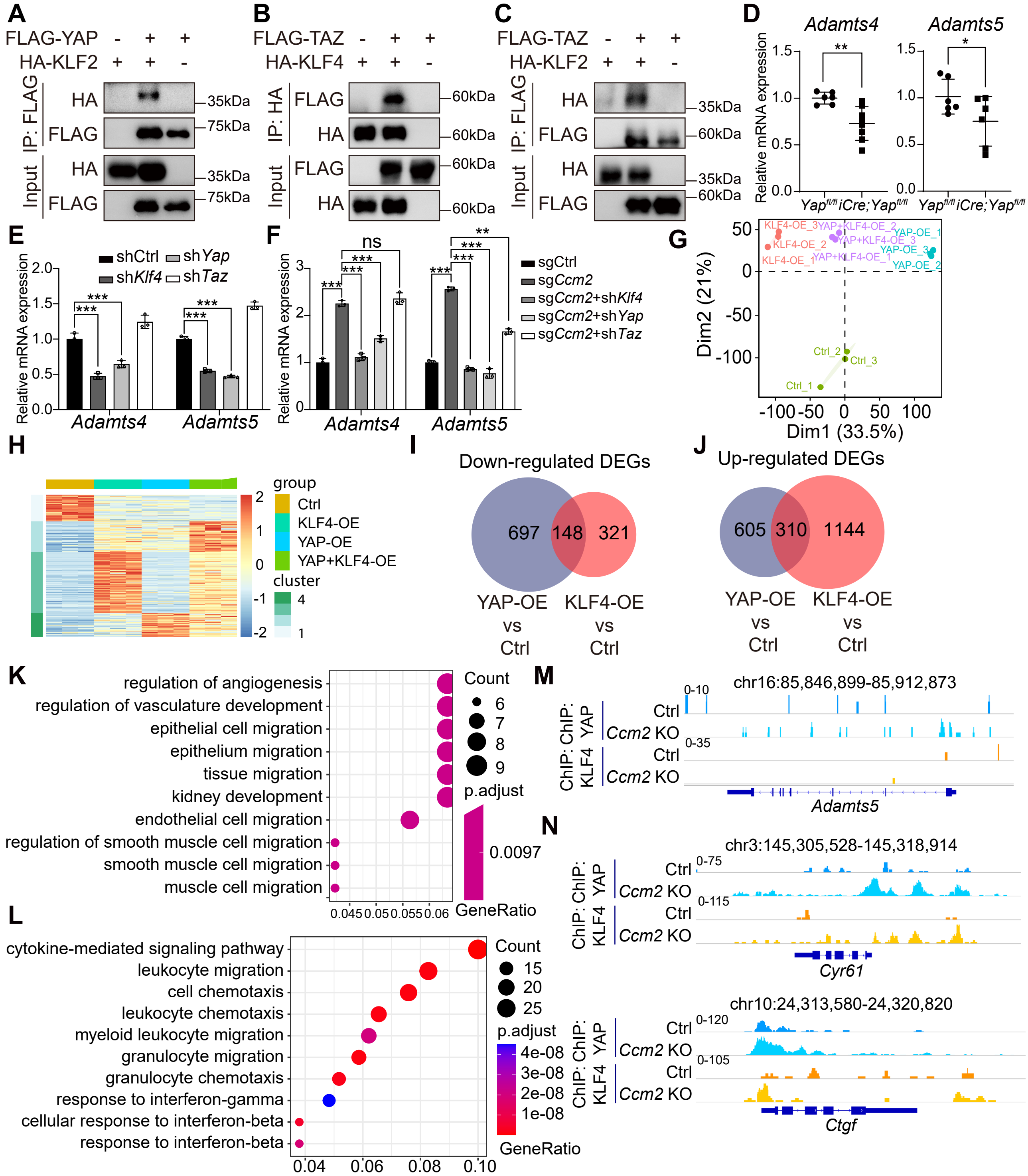
A
FLAG-YAP - + +
HA-KLF2 + + -
IP: FLAG
HA
FLAG
Input
35kDa
75kDa
B
FLAG-TAZ - + +
HA-KLF4 + + -
IP: HA
60kDa
C
FLAG-TAZ - + +
HA-KLF2 + + -
D
Adamts4
Adamts5
Relative mRNA expression
Yap fl/fl
iCre;Yap fl/fl
E
shCtrl
shYap
shKlf4
shTaz
F
ns
sgCtrl
sgCcm2
sgCcm2+shKlf4
sgCcm2+shYap
sgCcm2+shTaz
G
Dim2 (21%)
Dim1 (33.5%)
KLF4-OE_1
KLF4-OE_2
KLF4-OE_3
YAP+KLF4-OE_1
YAP+KLF4-OE_2
YAP+KLF4-OE_3
YAP-OE_1
YAP-OE_2
YAP-OE_3
Ctrl_1
Ctrl_2
Ctrl_3
H
group
Ctrl
KLF4-OE
YAP-OE
YAP+KLF4-OE
cluster
I
Down-regulated DEGs
697
148
321
YAP-OE vs Ctrl
KLF4-OE vs Ctrl
J
Up-regulated DEGs
605
310
1144
K
regulation of angiogenesis
regulation of vasculature development
epithelial cell migration
epithelium migration
tissue migration
kidney development
endothelial cell migration
regulation of smooth muscle cell migration
smooth muscle cell migration
muscle cell migration
Count
p.adjust
0.0097
GeneRatio
L
cytokine-mediated signaling pathway
leukocyte migration
cell chemotaxis
leukocyte chemotaxis
myeloid leukocyte migration
granulocyte migration
granulocyte chemotaxis
response to interferon-gamma
cellular response to interferon-beta
response to interferon-beta
M
chr16:85,846,899-85,912,873
ChIP: YAP
ChIP: KLF4
Ctrl
Ccm2 KO
Adamts5
N
chr3:145,305,528-145,318,914
Cyr61
chr10:24,313,580-24,320,820
Ctgf

Figure S9. YAP and TAZ interact with KLF2 and KLF4.

A. Co-IP analysis of YAP–KLF2 interaction. HEK293T cells were transiently co-transfected with FLAG-YAP and HA-KLF2. FLAG-YAP was immunoprecipitated, and HA-KLF2 was detected by immunoblotting. **B.** Co-IP analysis of TAZ–KLF4 interaction. HEK293T cells were transiently co-transfected with FLAG-TAZ and HA-KLF4. HA-KLF4 was immunoprecipitated, and FLAG-TAZ was detected by immunoblotting. **C.** Co-IP analysis of TAZ–KLF2 interaction. HEK293T cells were transiently co-transfected with FLAG-TAZ and HA-KLF2. FLAG-TAZ was immunoprecipitated, and HA-KLF2 was detected by immunoblotting. **D.** RT-qPCR analysis of *Adamts4* and *Adamts5* mRNA levels in primary MBMECs from adult *Yap*$^{fl/fl}$ and *iCre*;*Yap*$^{fl/fl}$ mice (n=6 per genotype). **E and F.** RT-qPCR analysis showing that *Yap* knockdown reduces *Adamts4* and *Adamts5* mRNA levels in both WT (E) and *Ccm2*-knockout (F) bEnd.3 cells (n=3). **G.** PCA plot showing the distinct clustering patterns among experimental groups. bEnd.3 cells were generated to overexpress YAP (YAP-OE), KLF4 (KLF4-OE),or both (YAP+KLF4-OE). Total RNA was extracted for RNA-seq analysis. **H.** Heatmap showing distinct expression patterns among the indicated groups. **I.** Venn diagram showing 148 DEGs downregulated by both YAP-OE and KLF4-OE. **J.** Venn diagram showing 310 DEGs upregulated by both YAP-OE and KLF4-OE. **K.** GO enrichment analysis of the co-downregulated genes shown in (I). **L.** GO enrichment analysis of the co-upregulated genes shown in (J). **M.** CUT&Tag genomic tracks showing YAP and KLF4 binding at the *Adamts5* locus in primary MBMECs from adult *Ccm2*$^{fl/fl}$ and *iCre*;*Ccm2*$^{fl/fl}$ mice. **N.** CUT&Tag genomic tracks showing YAP and KLF4 binding at the *Cyr61* and *Ctrgf* loci in primary MBMECs from adult *Ccm2*$^{fl/fl}$ and *iCre*;*Ccm2*$^{fl/fl}$ mice. Statistical analyses were performed using unpaired Student's t-test (D) or two-way ANOVA followed by Tukey's multiple comparisons test (E, F). Data are shown as means ± SEM; *$P < 0.05$; **$P < 0.01$; ***$P < 0.001$.

Fig S10

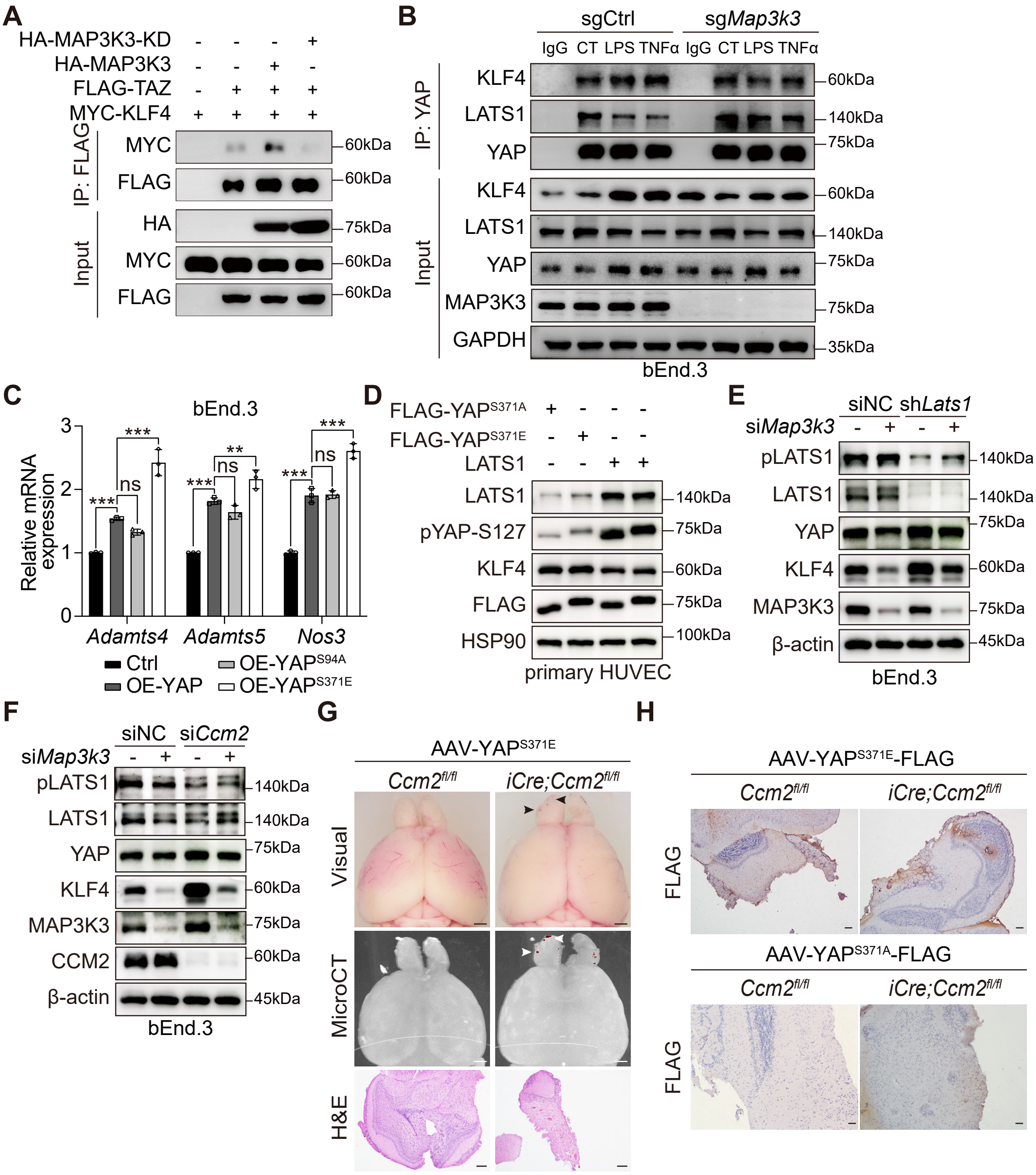

A
HA-MAP3K3-KD
HA-MAP3K3
FLAG-TAZ
MYC-KLF4
IP: FLAG
MYC
FLAG
Input
HA
MYC
FLAG
60kDa
75kDa
B
sgCtrl
sgMap3k3
IgG CT LPS TNFα IgG CT LPS TNFα
IP: YAP
KLF4
LATS1
YAP
Input
MAP3K3
GAPDH
140kDa
35kDa
bEnd.3
C
bEnd.3
Relative mRNA expression
Adamts4
Adamts5
Nos3
Ctrl
OE-YAP
OE-YAP S94A
OE-YAP S371E
ns
D
FLAG-YAP S371A
FLAG-YAP S371E
pYAP-S127
HSP90
100kDa
primary HUVEC
E
siNC
shLats1
siMap3k3
pLATS1
β-actin
45kDa
F
siCcm2
CCM2
G
AAV-YAP S371E
Ccm2 fl/fl
iCre;Ccm2 fl/fl
Visual
MicroCT
H&E
H
AAV-YAP S371E-FLAG
AAV-YAP S371A-FLAG

Figure S10. Activation of YAP at serine 371 by MAP3K3 facilitates CCM pathogenesis.

A. Co-IP analysis of KLF4-TAZ interaction in the presence of MAP3K3 or MAP3K3-KD. HEK293T cells were transiently co-transfected with MYC-KLF4, FLAG-TAZ and HA-MAP3K3 or HA-MAP3K3-KD. FLAG-TAZ was immunoprecipitated, and MYC-KLF4 was detected by immunoblotting. **B.** Co-IP analysis of KLF4-YAP interaction in LPS- or TNFα-treated *Map3k3* knockout bEnd.3 cells. Cells were treated with 100 ng/mL TNFα or 1 μg/mL LPS for 3 h. YAP proteins were immunoprecipitated, and KLF4 and LATS1 proteins were detected by immunoblotting. **C.** RT-qPCR analysis of *Adamts4*, *Adamts5* and *Nos3* mRNA levels in bEnd.3 cells expressing YAP^{S94A} or YAP^{S371E} mutants (n=3). **D.** Western blot analysis of pYAP-S127 levels in primary HUVEC transduced with lentivirus expressing LATS1 and FLAG-YAP^{S371A} or FLAG-YAP^{S371E}. **E.** Western blot analysis of KLF4 and YAP protein levels in WT and *Lats1*-knockdown bEnd.3 cells treated with si*Map3k3* or control siRNA. **F.** Western blot analysis of KLF4 and YAP protein levels in WT and *Ccm2*-knockdown bEnd.3 cells treated with si*Map3k3* or control siRNA. **G.** Representative visual, micro-CT and H&E images of brains collected 30 days after intracranial injection of YAP^{S371E}-expressing AAV virus into adult $Ccm2^{fl/fl}$ and *iCre*;$Ccm2^{fl/fl}$ mice. Arrows indicate hemorrhage sites. Scale bars: 1 mm (visual and micro-CT images); 50 μm (H&E images). **H.** DAB staining for the FLAG tag confirmed successful infection of the mouse brain by AAV- YAP^{S371E}-FLAG or YAP^{S371A}-FLAG. Scale bars: 50 μm. Statistical analyses were performed using two-way ANOVA followed by Tukey's multiple comparisons test (C). Data are shown as means ± SEM; n.s., not significant, $P > 0.05$; $^{**}P < 0.01$; $^{***}P < 0.001$.

Fig S11

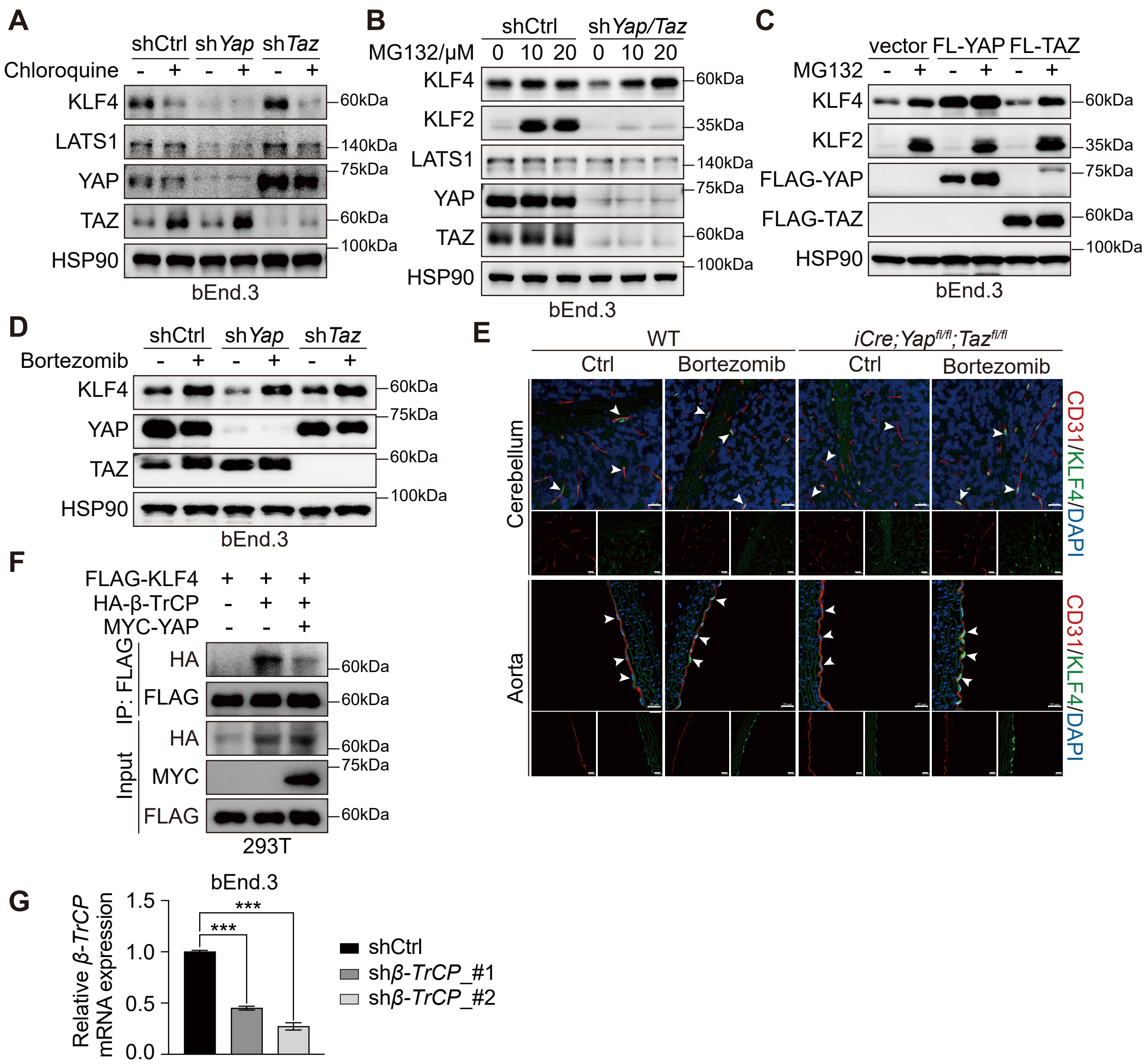

A
shCtrl shYap shTaz
Chloroquine - + - + - +
KLF4
LATS1
YAP
TAZ
HSP90
60kDa
140kDa
75kDa
60kDa
100kDa
bEnd.3
B
shCtrl shYap/Taz
MG132/μM 0 10 20 0 10 20
KLF4
KLF2
LATS1
YAP
TAZ
HSP90
60kDa
35kDa
140kDa
75kDa
60kDa
100kDa
bEnd.3
C
vector FL-YAP FL-TAZ
MG132 - + - + - +
KLF4
KLF2
FLAG-YAP
FLAG-TAZ
HSP90
60kDa
35kDa
75kDa
60kDa
100kDa
bEnd.3
D
shCtrl shYap shTaz
Bortezomib - + - + - +
KLF4
YAP
TAZ
HSP90
60kDa
75kDa
60kDa
100kDa
bEnd.3
E
WT
iCre;Yapfl/fl;Tazfl/fl
Ctrl
Bortezomib
Ctrl
Bortezomib
Cerebellum
Aorta
CD31/KLF4/DAPI
CD31/KLF4/DAPI
F
FLAG-KLF4 + + +
HA-β-TrCP - + +
MYC-YAP - - +
IP: FLAG
HA
FLAG
Input
HA
MYC
FLAG
60kDa
60kDa
60kDa
75kDa
60kDa
293T
G
bEnd.3
Relative β-TrCP mRNA expression
1.5
1.0
0.5
0.0
***
***
shCtrl
shβ-TrCP_#1
shβ-TrCP_#2

Figure S11. Proteasome inhibition restores KLF4 expression in *Yap*-deficient endothelial cells.

A. Western blot analysis of KLF4 protein levels in WT, *Yap* and *Taz* knockdown bEnd.3 cells treated with Chloroquine (CQ)-treated (20 μM) for 4 h before harvest. **B.** Western blot analysis of KLF4 protein levels in WT, *Yap* knockdown and *Taz* knockdown bEnd.3 cells treated with MG132 (20 μM) for 4h. **C.** Western blotting analysis of KLF4 protein levels in WT, YAP-overexpressing, and TAZ-overexpressing bEnd.3 cells treated with MG132 (20 μM) for 4h. **D.** Western blot analysis of KLF4 protein levels in WT, *Yap*-knockdown and *Taz*-knockdown bEnd.3 cells treated with bortezomib (10 μM) for 24 h. **E.** Immunofluorescence staining of KLF4 (green) and CD31 (red) in the cerebellum or aortic endothelium from adult WT and *iCre*; $Yap^{fl/fl}$;$Taz^{fl/fl}$ mice. Mice received three intraperitoneal injections of 100 μg tamoxifen and then two intravenous injections of 20 μg bortezomib or vehicle within 24 hours. Nuclei were counterstained with DAPI (blue). n = 3 mice per genotype. Scale bars: 20 μm. **F.** Co-IP analysis showing that YAP inhibits KLF4–β-TrCP interaction. HEK293T cells were transiently co-transfected with FLAG-KLF4, HA-β-TrCP, and MYC-YAP. FLAG-KLF4 was immunoprecipitated, and HA-β-TrCP was detected by immunoblotting. **H.** RT-qPCR analysis of *β-TrCP* mRNA levels in *β-TrCP*-knockdown bEnd.3 cells (n=3). Statistical analyses were performed using one-way ANOVA followed by Dunnett's multiple comparison test (H). Data are shown as means ± SEM, ***$P < 0.001$.

Supplementary Methods

Evans blue extravasation

Adult mice were injected with Evans blue solution (Sangon Biotech) (2% in sterile PBS, 10 μL/g body weight) via tail vein. The mice were euthanized 12 h, 24 h or 48 h after injection, and brain tissues were harvested after intracardiac perfusion with PBS. Evans blue was extracted using 1 mL of formamide at 55°C for 16 h, followed by spectrophotometric measurement of absorbance at 630 nm.

Inflammatory stimulation

LPS (Sigma) and TNFα (Peprotech) were initially dissolved in PBS and subsequently diluted to the indicated concentrations in a complete DMEM medium. Primary MBMECs were treated with 500 ng/mL TNFα or 1 μg/mL LPS in a time course manner.

Neonatal mice were injected intraperitoneally with 20 μg of tamoxifen on the first (P1) and third (P3) days after birth. After intraperitoneal injection of 5 μg LPS at P6, the brains were harvested on day P12.

Immunofluorescence

bEnd.3 cells or primary MBMECs were seeded onto coverslips pretreated with 0.1% gelatin, and fixed with 4% PFA for 20 min, followed by permeabilization with 0.5% Triton X-100 (in PBS) for 5 min. Subsequently, the cells were blocked with 3% BSA in PBST containing 0.1% Tween-20 for 1 h and incubated with primary antibodies diluted in 3% BSA/PBST at 1:100 for 3 h at room

temperature. After three washes with PBST, the cells were incubated with the corresponding secondary antibodies in 3% BSA/PBST for 1 h. Nuclei were stained with DAPI for 10 min. Images were acquired by confocal microscopy.

Western blotting

Cell lysates were resolved by performing 10% sodium dodecyl sulfate-polyacrylamide gel electrophoresis (SDS-PAGE) and the resolved proteins were subsequently transferred to polyvinylidene difluoride (PVDF) membranes (Millipore). The membranes were then blocked with 5% nonfat milk/TBST or 5% BSA/TBST for 1 h before incubating with primary antibodies (Table S2) at 4 ℃ overnight. After washing three times in TBST, the membranes were incubated with the corresponding HRP-conjugated secondary antibodies for 1 h. Immunoreactive signals were detected using enhanced chemiluminescence (ECL) substrate. Images were captured by ChemiScope5300 (Clinx). HSP90, GAPDH and β-actin were used as the internal loading controls for protein normalization in western blotting assay.

RT-qPCR

Total RNA was isolated using RNAiso Plus (Takara) and reverse-transcribed to cDNA using 5x PrimeScript RT master mix (Takara). qPCR was performed using Quantagene q225 (Kubo Technology Co., Ltd.) with PerfectStart Green qPCR SuperMix (2x) (TRans). Primers are listed in Table S3. *HSP90* and *GAPDH* were used as the reference genes.

RNA-seq

Total RNA was extracted from bEnd.3 cells overexpressing YAP, KLF4, and co-overexpressing YAP and KLF4 using RNAiso Plus. cDNA libraries were generated using Hieff NGS® Ultima Dual-mode mRNA Library Prep Kit for Illumina® (Cat#12301, Yeasen Biotechnology) following the manufacturer's recommendations. The libraries were then sequenced on the DNBSEQ-T7 platform (Geneplus-Shenzhen). Raw sequencing data were deposited in the GEO database (GSE280200). Raw reads were trimmed to remove adapters and low-quality sequences using Trim Galore (version 0.6.6) (https://www.bioinformatics.babraham.ac.uk/projects/trim_galore/) with the parameters '-q 20 --paired'. Then trimmed reads were uniquely mapped to the mouse reference genome (mm9) using STAR (version 2.7.7a) [1]. The output SAM files were converted to sorted BAM files using SAMtools (version 1.11) [2]. Expression matrixes were counted by featureCounts (version 2.0.1) [3] and normalized as fragments per kilobase per million mapped reads (FPKM) by StringTie (version 2.1.7) [4]. R package DESeq2 (version 1.34.0) [5] was used to identify genes with different expression levels, which were selected based on the threshold '$|\log2 (\text{foldchange})| > 1$' and '$FDR < 0.05$'. Gene Ontology (GO), Gene Set Enrichment Analysis (GSEA) and Kyoto Encyclopedia of Genes and Genomes (KEGG) pathway enrichment analyses were performed using clusterProfiler (version 4.2.2) [6].

CUT&Tag assay

Primary MBMECs from *Ccm2*$^{fl/fl}$ and *iCre;Ccm2*$^{fl/fl}$ mice were processed for CUT&Tag according to the protocol [7]. Briefly, the cells were washed and lightly cross-linked with formaldehyde to stabilize the nuclear structures. After binding to Concanavalin A-coated Magnetic Beads (Novoprotein Scientific Inc., N251), the cells were gently mixed with mouse anti-YAP or rabbit anti-KLF4 antibodies, followed by incubation with secondary antibody (goat anti-mouse IgG H&L, goat anti-rabbit IgG H&L, 1:100, Abcam). After tag-mentation by binding to the pG-Tn5 adapter complex (1:100), DNA was extracted using phenol/chloroform method, and PCR amplification was conducted to prepare the library. All libraries were sequenced using a NovaSeq 6000 (Illumina) by Beijing Novogene Bioinformatics Technology after DNA purification with Ampure XP beads (Beckman Coulter, A63881). Raw sequencing data were deposited in the GEO database (GSE280201).

Raw reads were trimmed to remove adapters and low-quality sequences using Trim Galore (version 0.6.6) (https://www.bioinformatics.babraham.ac.uk/projects/trim_galore/) with the parameters '-q 20 --paired'. Then trimmed reads were uniquely mapped to the mouse reference genome (mm9) using Bowtie2 (version 2.4.2) [8]. The output SAM files were converted to sorted BAM files using SAMtools (version 1.11). Genome coverage bigwig files were generated by BEDtools (version 3.5.0) [9] genomecov and bedGraphToBigWig (version 4) (https://www.encodeproject.org/software/bedgraphtobigwig/) with the

parameter '-scale 10,000,000/ (the number of mapped reads)'. Heatmaps were generated by deepTools (version 3.5.0) [10] computeMatrix and plotHeatmap. The Pearson product-moment correlation was calculated using a 1 Kb sliding window across the whole genome.

Supplementary Tables

Table S1: Guide RNA, shRNA, and siRNA sequences

	**Guide RNA**	
Ccm2	mouse	AATCCTGCATTTCATAGACA
Map3k3	mouse	TGAACAAGAGGCATTAGACT
	**shRNA**	
*Lats1*_#1	mouse	CCTATTCAACAGCCCGTGAAA
*Lats1*_#2	mouse	GCAACATTCAATTAACCGAAA
Yap1	mouse	GCTGCCACCAAGCTAGATAAT
Taz	mouse	CGCCAGAGATACTTCCTTAAT
Klf4	mouse	ACCTATACCAAGAGTTCTCAT
*β-TrCP*_#1	mouse	CCGCCTCCAGTTTGATGAATT
*β-TrCP*_#2	mouse	GCCAGGCTTTGCATAAACCAA
	**siRNA**	
*Ccm2*_#1	mouse	GGACAGTCAGCACTTTGAA
*Ccm2*_#2	mouse	GCTACATGAGTACCGCAAT
Yap1	mouse	GCCACCAAGCUAGAUAAAGAATT
Klf4	mouse	ACCUAUACCAAGAGUUCUCAUTT
Map3k3	mouse	UCACCAUCUUUAUGGAGUAUATT
CCM2	human	GCAUUUCAUAGACAAUGCAAATT

Table S2: Antibody information

Antibody	Company	Cat No.
Rabbit Anti-Phospho-LATS1/2	Cell Signaling Technologies	8654S
Rabbit Anti-LATS1	Cell Signaling Technologies	3477S
Mouse Anti-YAP	Santa Cruz	sc-101199
Rabbit Anti-YAP	Cell Signaling Technologies	4912S
Rabbit Anti-Phospho-YAP (S127)	Cell Signaling Technologies	13008S
Rabbit Anti-TAZ	Cell Signaling Technologies	72804S
Rabbit Anti-KLF4	Abcam	ab214666
Rabbit Anti-KLF4	Abcam	ab215036
Rabbit Anti-MAP3K3	Abcam	ab40756
Mouse Anti-NOS3	Abcam	ab76198
Rabbit Anti-ADAMTS4	Abclonal	A2525
Rabbit Anti-ADAMTS5	Abclonal	A2836
Rabbit Anti-CCM2	Cell Signaling Technologies	32463T
Rabbit Anti-HSP90	Proteintech	13171-1-AP
Rabbit Anti-GAPDH	Proteintech	60004-1-Ig
Rat Anti-HA	Roche	11867431001
Mouse Anti-FLAG	Sigma	F3165
Mouse Anti-MYC	BIOXCELL	BE0238
Rabbit Anti-α-Tubulin	Proteintech	11224-1-AP
Mouse Anti-SOX2	Proteintech	11064-1-AP
Rabbit Anti-VE-cadherin	Abcam	ab318152
Mouse Anti-ZO-1	Invitrogen	33-9100
Rabbit Anti-CD31	Cell Signaling Technologies	77699S
Rabbit Anti-CD31	Abcam	ab182981
Rabbit Anti-CCM1	Abcam	ab196025

Rabbit Anti-CCM3	Abclonal	A15400
Rabbit Anti-Phospho-P65	Cell Signaling Technologies	3033S
Rabbit Anti-pan-TEAD	Cell Signaling Technologies	13295S
Rabbit Anti-β-actin	Abclonal	AC026

Table S3: qPCR primers

Gene	Species	Forward primer	Reverse primer
Lats1	mouse	TGGGACAACCTCCTTTCTTGGC	TGAGGTCAGAGGCTTCAGGACT
Yap1	mouse	CCAGACGACTTCCTCAACAGTG	GCATCTCCTTCCAGTGTGCCAA
Klf4	mouse	TACCCCTCTCTCTTCTTCGGA	TTCCTCACGCCAACGGTTAGT
Taz	mouse	GTCACCAACAGTAGCTCAGATCC	GTTGCTGAGGAAGTCTTCTGGAG
Cyr61	mouse	GTGAAGTGCGTCCTTGTGGACA	CTTGACACTGGAGCATCCTGCA
Ctgf	mouse	TGCGAAGCTGACCTGGAGGAAA	CCGCAGAACTTAGCCCTGTATG
Adamts4	mouse	GAACGGTGGCAAGTATTGTGAGG	TTCGGTGGTTGTAGGCAGCACA
Adamts5	mouse	CTGCCTTCAAGGCAAATGTGTGG	CAATGGCGGTAGGCAAACTGCA
eNOS	mouse	CGCAAGAGGAAGGAGTCTAGCA	TCGAGCAAAGGCACAGAAGTGG
β-actin	mouse	CATTGCTGACAGGATGCAGAAGG	TGCTGGAAGGTGGACAGTGAGG
Gapdh	mouse	CATCACTGCCACCCAGAAGACTG	ATGCCAGTGAGCTTCCCGTTCAG

Ccm2	mouse	TCCCATGGAGAACGAGCCTG	TATAGTCGCTCAGCAGTCTGTC
Klf2	mouse	TGCGTACACACACAGGTGAGA	CAGGCTACATGTGTCGCTTCA
β-TrCP	mouse	GCAGTACGATGAGAGGGTGATC	CAGAACGGCTTCACAGTGGTGA

Reference

1. Dobin, A., C.A. Davis, F. Schlesinger, et al., STAR: ultrafast universal RNA-seq aligner. *Bioinformatics*. 2013; 29(1): 15-21.

2. Danecek, P., J.K. Bonfield, J. Liddle, et al., Twelve years of SAMtools and BCFtools. *Gigascience*. 2021; 10(2): 008.

3. Liao, Y., G.K. Smyth, and W. Shi, featureCounts: an efficient general purpose program for assigning sequence reads to genomic features. *Bioinformatics*. 2014; 30(7): 923-930.

4. Pertea, M., G.M. Pertea, C.M. Antonescu, et al., StringTie enables improved reconstruction of a transcriptome from RNA-seq reads. *Nat Biotechnol*. 2015; 33(3): 290-295.

5. Love, M.I., W. Huber, and S. Anders, Moderated estimation of fold change and dispersion for RNA-seq data with DESeq2. *Genome Biol*. 2014; 15(12): 550.

6. Yu, G.C., L.G. Wang, Y.Y. Han, and Q.Y. He, clusterProfiler: an R Package for Comparing Biological Themes Among Gene Clusters. *Omics*. 2012; 16(5): 284-287.

7. Kaya-Okur, H.S., D.H. Janssens, J.G. Henikoff, K. Ahmad, and S. Henikoff, Efficient low-cost chromatin profiling with CUT&Tag. *Nat Protoc*. 2020; 15(10): 3264-3283.

8. Langdon, W.B., Performance of genetic programming optimised Bowtie2 on genome comparison and analytic testing (GCAT) benchmarks. *BioData Min*. 2015; 8(1): 1.
9. Quinlan, A.R., BEDTools: The Swiss-Army Tool for Genome Feature Analysis. *Curr Protoc Bioinformatics*. 2014; 47: 11.12.11-11.12.34.
10. Ramirez, F., F. Dundar, S. Diehl, B.A. Gruning, and T. Manke, deepTools: a flexible platform for exploring deep-sequencing data. *Nucleic Acids Res*. 2014; 42(Web Server issue): W187-191.
